# A deliberative public engagement study on heritable human genome editing among South Africans: Study results

**DOI:** 10.1371/journal.pone.0275372

**Published:** 2022-11-28

**Authors:** Donrich Thaldar, Bonginkosi Shozi, Michaela Steytler, Gill Hendry, Marietjie Botes, Ntokozo Mnyandu, Meshandren Naidoo, Siddharthiya Pillay, Magda Slabbert, Beverley Townsend

**Affiliations:** 1 School of Law, University of KwaZulu-Natal, Durban, South Africa; 2 Institute for Practical Ethics, University of California San Diego, San Diego, California, United States of America; 3 Unaffiliated, South Africa; 4 Interdisciplinary Centre for Security, Reliability and Trust, Université du Luxembourg, Luxembourg, Luxembourg; 5 School of Management, Information Technology & Governance, University of KwaZulu-Natal, Durban, South Africa; 6 College of Law, University of South Africa, Pretoria, South Africa; 7 York Law School, University of York, York, United Kingdom; Bryant University, UNITED STATES

## Abstract

This paper reports the results of a public engagement study on heritable human genome editing (HHGE) carried out in South Africa, which was conducted in accordance with a study protocol that was published in this journal in 2021. This study is novel as it is the first public engagement study on HHGE in Africa. It used a deliberative public engagement (DPE) methodology, entailing inter alia that measures were put in place to ensure that potential participants became informed about HHGE, and that deliberations between the participants were facilitated with the aim of seeking consensus. A diverse group of 30 persons was selected to participate in the DPE study, which took place via Zoom over three consecutive weekday evenings. The main results are: Provided that HHGE is safe and effective, an overwhelming majority of participants supported allowing the use of HHGE to prevent genetic health conditions and for immunity against TB and HIV/Aids, while significant majorities opposed allowing HHGE for enhancement. The dominant paradigm during the deliberations was balancing health benefits (and associated improvements in quality of life) with unforeseen health risks (such as loss of natural immunity). The seriousness of a health condition emerged as the determining factor for the policy choice of whether to allow an application of HHGE. More generally, equal access to HHGE *qua* healthcare service featured as an important value, and it was uncontested that the South African government should allocate resources to promote scientific research into HHGE. These results are aligned with the policy principles for regulating HHGE in South Africa suggested by Thaldar et al. They call for urgent revision of South African ethics guidelines that currently prohibit research on HHGE, and for dedicated HHGE legal regulations that provide a clear and comprehensive legal pathway for researchers who intend to conduct HHGE research and clinical trials.

## I. Introduction

### i. Importance of this study

Since the advent of CRISPR-Cas9, several scholars, academic institutions and government entities have weighed in on the ethical, social and legal issues raised by genetic technologies and their potential use for heritable human genome editing (HHGE). A recurring theme that has emerged from the literature has been the desirability of a greater societal discourse on the potential use of HHGE to make heritable changes to prospective persons, with some even suggesting that ‘broad societal consensus’ must precede any clinical applications of HHGE [1]. Similar sentiments have been echoed in the growing literature on the governance of HHGE. A number of institutions tasked with advising states on developing policy in this area have emphasised the need for a broader societal debate through public engagement or consultation [2, 3]. Calls for public participation in scientific and technological developments such as these are rooted in the understanding that decisions about the course of technological development should be made by society as a whole—rather than by just a small group of experts as traditionally is the case [1].

Although there is a convincing case for public engagement, one may be sceptical of an endeavour to attain so-called ‘societal consensus’, on the grounds that it is clearly impossible to obtain universal agreement on any issue relating to HHGE. Does this mean that any endeavour to engage in public engagement is fruitless? Not at all. While any attempts at achieving a true ‘societal consensus’ are bound to fail due to a litany of practical limitations, there is nevertheless much to be gained by a wider public discourse on the governance of novel technologies. As Townsend explains, even where public engagement may not be capable of securing clear consensus, it can have benefit in the context of developing effective governance for HHGE, by giving democratic legitimacy to HHGE policy and securing public trust in genetic technologies [4]. The potential role public engagement could play in policy development relating to HHGE was outlined in a 2021 opinion by the European Group on Ethics:

Against the background of these far-reaching questions about concepts of humanness and naturalness and their ethical dimensions, it is clear that there is no one scientific, unambiguous and thus binding answer as to what the relation between the genome of a human embryo and humanness and naturalness is, and what ethical orientation this can provide. Rather, there is a need for a broad, inclusive and nuanced social debate on the foundations of our view (or indeed many possible views) of humanity, which takes all perspectives into account and brings them into discussion [5].

In addition to the critical role public engagement can play in helping policymakers identify where lines should be drawn on the plethora of ethical issues raised by HHGE, the process of public engagement also serves a number of pragmatic functions. For instance, public engagement can provide much needed insight on how to ‘understand and mitigate the risks’ that the public perceives as important, as well as ‘allay public fears arising from the use of genome editing technology’ [6]. Engaging with the public’s feelings about technology (be they negative or positive) is important because of how public perceptions of technology tend to be driven by misconceptions of the science underlying that technology [7–9]. Through public engagement, the public can have their views engaged with, which creates a platform for both baseless fear and unbridled optimism based on misconceptions to be attended to. Ergo, even if true ‘societal consensus’ is beyond the realm of possibility, public engagement can have an important role to play in the future governance of HHGE. These gains make the endeavour to engage in a broad societal discourse worthwhile. This then raises the question of how public engagement can best be executed in order for policymakers to benefit from the insights gained from such public engagement. This paper describes an approach to answering this question based on a public engagement initiative carried out in South Africa using a *deliberative* public engagement (DPE) methodology [10]. This study is novel as it is the first public engagement study on HHGE in Africa.

The main characteristics of the DPE methodology that differentiate it from other public engagement methodologies are: (a) participants are not people with vested interests in the topic but are recruited through sortition; (b) participants are provided with balanced information about the topic to ensure informed deliberation; and (c) deliberation is facilitated and consensus-seeking [11–13]. Each of these features make DPE an attractive public engagement methodology to use in the context of new genomic technologies such as CRISPR, because: (a) it ensures that participants are truly ‘members of the public’, and not stakeholders driven by personal agendas such as financial interests; (b) entails educating members of the public about novel technologies ensuring that the views expressed are not driven by ignorance or misconceptions about the technology; and (c) encourages participants to reflect on and critically engage with their values, ensuring that results do not simply reflect unexamined preferences (which may be driven by biases). Building on the strengths of the DPE methodology, this study aimed to answer the question: What is the well informed, considered public opinion on HHGE in South Africa?

In this paper we describe this study and consider its broader implications for the governance of HHGE in South Africa. We begin by describing some core terminology, followed by a description of the materials and methods. We then provide an overview of the results, with a brief discussion of the dominant themes that emerged from the study. We highlight what we observe as the most critical finding—the extent to which local context has a bearing on public opinion.

### ii. Terminology

The bioethical debate on HHGE inherited the therapy-enhancement dichotomy from related debates of previous generations [14]. Typically, the term ‘therapeutic’ HHGE refers to the correction of a genetic defect in germ cells, with the aim of the genome-edited individual being born with a ‘normal’ genome. The term ‘non-therapeutic’ HHGE refers to the modification of a normal genome in germ cells, with the aim being that the genome-edited individual is born with an ‘enhanced’ genome [15]. However, ‘therapeutic’ can also be understood more broadly as anything that is done for reasons of health. This would mean that the modification of a normal genome for immunity against disease constitutes an intersectional category that can be construed as both *therapeutic* HHGE (done to bestow a health benefit on the genome-edited individual) and *enhancement* HHGE (the genome was not returned to a ‘normal’ state, but instead a ‘normal’ genome was ‘enhanced’). To avoid confusion, in this article we refer to this intersectional category as ‘HHGE for immunity’. When we refer to ‘HHGE for enhancement’, we refer to those uses of HHGE that are *solely* enhancement (not done to prevent a genetic health condition or to make the genome-edited individual immune to disease).

### iii. Overview of public engagement research on HHGE

Public engagement may broadly be described as ‘processes and initiatives focused on enabling public participation in the responsible innovation and development of new technologies, including the management and assessment of technological risks’ [16]. As alluded to above, despite there being no shortage of support for public engagement on HHGE, there have been very few concrete proposals on how best to approach the process of discerning the public’s opinions. This is partially because public opinion is a vast concept, which can be conceptualised in a number of ways depending on the ‘how’ and ‘when’ of the public engagement exercise used to obtain the public’s views [1]. There are various modalities of public engagement, which are defined by the varying goals that each of these approaches attempts to achieve [16]. That said, when people speak of public opinion on genome editing, it is usually with reference to the ‘polled public’ [1], which is constituted of a poll of the views of a group of individuals who are (generally) representative of a particular country [17]. This is because engagement with the public on science has traditionally been limited to the polled public—as has been the case in most public engagement studies on HHGE in recent years [18].

Analysis of public engagement studies on HHGE shows that the general public around the world is open to at least some applications of HHGE–predominantly applications that are of a ‘therapeutic’ nature, such as HHGE for preventing a child from inheriting a genetic health condition [19–22]. For example, in a 2017 survey commissioned by the Royal Society, 76% of adults in the United Kingdom felt positively about genome editing ‘to correct a disorder so that the correction would also be inherited by any children that person has’ [23]. Similarly, a poll by the Associated Press-NORC Center for Public Affairs Research (AP-NORC) reported that 71% of Americans favoured HHGE for preventing a child being born with incurable and potentially fatal diseases [24]. Similar levels of support for HHGE to ‘treat’ a serious genetic condition—by preventing a child being born with said disease—were reported in the most recent PEW Research Center study of representative samples of the general public in 20 different countries [25].

On the other hand, using HHGE to edit for enhanced genetic traits is generally perceived by the public as unnecessary and undesirable [21, 26]. The Royal Society study found little support for HHGE for enhanced ‘cosmetic’ traits (such as height) in the UK [23]. Similarly, in the United States, a relatively small portion of Americans favoured using HHGE to enhance traits such as intelligence, athletic ability, eye colour and height, according to the AP-NORC study [24]. Although opinion polls generally show greater support for applications of HHGE technologies for preventing genetic health conditions over these kinds of applications [20]. it is worth noting that in many cases so-called genetic enhancements have been well received where there was a perceived good reason for them. For instance, the PEW Research Centre reported a median of 60% of participants in all 20 countries stating that it was appropriate to use HHGE ‘to reduce the risk of a serious disease that could occur over the course of the child’s lifetime’—although significantly fewer (a median of 14%) felt the same about using HHGE to make a prospective child more intelligent [25]. Similarly, in the AP-NORC study, 67% of Americans favoured HHGE to reduce the risk of potentially life-threatening diseases such as cancer [24]. These sentiments also apply to using HHGE to prevent genetic health conditions that are not life threatening, but which are nevertheless viewed as causing serious impairment or disability. For instance, 65% of Americans favoured HHGE for ‘a non-fatal condition that a child would inherit, such as blindness’ [24].

Interestingly, it seems that public opinion favours using HHGE to prevent health conditions—even if those conditions are not genetic—provided they are perceived as being sufficiently serious. Howell et al, in their systemic review of representative public engagement studies, note that there are generally high levels of acceptance for HHGE applications aimed at preventing the occurrence of even non-genetic health conditions (relative to other applications that may be classed as a genetic enhancement), likely because these applications are viewed by the general public as being analogous to using HHGE to prevent genetic health conditions [19]. In one study by Jedwab et al of 1537 participants across 67 countries, over 65% of participants favoured using HHGE to impart resistance for serious infectious diseases [22].

In conclusion, the current literature on public engagement in respect of applications of HHGE suggests that the public is generally accepting—provided it is to prevent the inheritance of genetic health conditions, or to prevent serious health conditions from occurring. Applications of HHGE for purposes outside of this are generally not viewed favourably.

### iv. Why deliberative public engagement?

The current literature provides insights, including the results of public opinion polls, that can be of value to the project of governance of HHGE. However, public opinion polls on HHGE have primarily focused on the US and the UK, with no similar study published in the African context, and few elsewhere in the Global South [19]. Given this dearth of research on public opinion in many jurisdictions (with some having had no research conducted there, and others very little), it is unclear to what extent the findings of the US and UK studies could be viewed as generally applicable across the globe. Indeed, a breakdown of the Pew Research Center’s research suggests that regional viewpoints may vary significantly: While 86% of people in Japan felt that HHGE to increase intelligence was a misuse of technology, 64% of people in India supported it [25]. This is just one example that illustrates the extent to which perspectives on value-laden issues such as acceptable uses of technology may vary significantly. There is clearly an imperative for public engagement initiatives for countries outside of the Global North, if such countries hope to develop effective policies on HHGE that speak to the values of their people. That being the case, it is also important to engage critically with the questions of how public engagement should be best carried out, in order to guide policy development on HHGE.

There is reason to be sceptical of using the current public engagement data as a baseline for governance. This is because of the extent to which these approaches tend to conceptualise public opinion as a congregation of views and opinions expressed by individuals in isolation. As Adashi et al point out, with polling: ‘individual responses are unlikely to have benefitted from information, reflection, and judgment’[1]. Ergo, to regulate HHGE as aligned with public opinion (as represented in polling data) would arguably be to make policy decisions based on the uninformed, unconsidered views of the majority, as gleaned through a representative sample [27, 28]. In contrast, Adashi et al describe expert-mediated deliberation as a space in which ‘long-held views of a pluralistic society may be revised through contestatory civic engagement’ that facilitates the development of an ‘enhanced state of mutual understanding’ [1].

The case against using the views of a majority group in a representative sample as a baseline for governance is reinforced when viewing HHGE through the paradigm of human rights. As scholars have pointed out, the extent to which several potential applications of HHGE touch on widely recognised fundamental rights and freedoms means that in liberal democracies, the opinions of a majority (or even all) of society would not be a legitimate basis upon which the exercise of these rights and freedoms can be limited [6, 15, 29, 30]. What may, however, provide a democratically legitimate basis for limitation, are policies based on *deliberative engagement* aimed at obtaining the well-informed, considered opinions of members of a community, which are expressions of the values of that country.

DPE has played a pivotal role in policy development for novel technologies in several countries [17]. In our view, a similar approach should be taken for developing governance for the use of HHGE technologies. The need for a DPE approach to public engagement on HHGE is premised on the novelty of technologies that can be challenging to properly comprehend, even for a generally knowledgeable person. As Schefuele et al point out, the deliberative process—during which individuals can engage with others, think through complex ideas, and hear contrary positions—is necessary because innovations such as CRISPR-Cas9 ‘demand new and more effective infrastructures for citizen engagement that go beyond classical modalities of civic participation’ [16].

There is growing support for the deliberative engagement approach to public engagement in the context of HHGE. An example of this is the number of scholars that have expressed support for ‘deliberation by a global citizens’ assembly’ [31]. This global citizens’ assembly will be a planned public engagement exercise, envisioned as a non-governmental platform for deliberation on genome editing by a group of randomly selected non-expert participants from various regions of the world. As proponents of this project point out, one of the main benefits of a deliberative approach to public engagement is that it provides an opportunity for informing the lay public about the science of genome editing through a process mediated by experts, and also for getting insight on the considered opinions of the public, rather than knee-jerk reactions [31]. For these reasons, we decided on a DPE approach to public engagement in the South African context. In the following section, we elaborate on the methodology used in this study. Finally, we conclude by elaborating on the implications of the findings of this study on the governance of HHGE in South Africa.

## II. Materials and methods

Ethical approval was obtained from the Humanities and Social Sciences Research Ethics Committee of the University of KwaZulu-Natal (protocol reference number HSSREC/00002595/2021, as amended). Informed consent was provided in writing, as detailed in our discussion below. Our study protocol was published in this journal [10]. Before conducting the study, we organised a pilot study to test and, where necessary, refine our study protocol. Below, we briefly review our study protocol and highlight areas where we amended or supplemented it.

### i. Participants

Our aim was to compose a group of participants in such a way as to (a) make meaningful deliberation practically possible, which means that we limited the number of participants to 30; and (b) be as inclusive as possible on the demographic metrics of race, gender, educational attainment, age, and religion/belief. These are commonly used demographic categories for recruiting participants from diverse backgrounds with a view to increasing the likelihood and prevalence of divergent views and attitudes [10]. Given (a) and (b), we did not aim to recruit a representative sample of the population.

Participants were recruited in the following steps: (Step 1): We launched a Facebook advertisement campaign among people living in South Africa. (Step 2): Interested candidates could click through to our project website to register. A total of 2474 candidates registered on the project website. (Step 3): After a registered candidates provided informed consent for eligibility screening by submitting an online form, they were requested to answer an online questionnaire to determine their eligibility. The eligibility criteria were: (a) citizenship of South Africa, (b) being of the age of majority (18 years), (c) willingness to learn more about genome editing and the associated ethical debates, (d) willingness to form one’s own opinions on the various uses of genome editing and to discuss one’s opinions on genome editing with others, (e) willingness to participate in three evenings of online meetings that would be recorded for research purposes, and (f) having sufficient internet and Zoom access. A total of 1205 of the registered candidates were eligible to participate in the study. (Step 4): Eligible candidates were provided with access to resource material and requested to take an online entrance exam to assess their comprehension of the resource material. Only those candidates who passed the entrance exam and provided informed consent for study participation by submitting an online form became part of a pool of potential participants. The entrance exam was only passed if all its questions were answered correctly. After taking the exam, candidates received their overall score only, not question-specific scores. Anticipating that unsuccessful candidates would wish to retake the exam a few times in order to pass, we placed no limit on the number of times a candidate could take the entrance exam. However, what we did not expect was that some candidates would use this arrangement to retake the exam many times—even over 100 times—before getting all the questions correct. Given that the purpose of the exam was to ensure that all our participants had a good understanding of the topic (and did not just exploit the system), we decided to limit our pool of 127 potential participants to those candidates who passed the exam in ten or fewer attempts. This provided us with a pool of 70 potential participants. (Step 5): From this pool, 30 participants were selected such that there was a group of participants that was as inclusive as possible on the demographic metrics of race, gender, educational attainment, age, and religion/belief. The categories within race (Black African, Coloured, Indian, White) and gender (male, female) are the standard categories used in South Africa for these metrics. For convenience, we categorised age in decades, and grouped ages 18 and 19 with the twenties’ category. To combine the aim of optimising inclusivity with our commitment to random participant selection, we used the following methodology in selecting the 30 participants:

1. Within each demographic metric: (a) the candidates were categorised according to their selected category; (b) the average number of candidates per category was calculated using only the categories containing candidates; and (c) categories that contained fewer than the average number of candidates were identified. There were 15 such less populous categories.
2. Within each of these less populous categories, a participant was selected at random. Once a candidate was selected, the candidate was removed from the pool of available candidates to avoid overlap. This step was repeated once. During the second round, one less populous category did not have any candidates remaining in the pool. As such, we had 29 participants selected at this stage.
3. To fill the last position, we decided to balance the gender composition that was 14 women and 15 men by selecting a woman at random.

We suggest that this methodology is useful to ensure that candidates from less populous categories in the demographic metrics are included in the eventual group of participants. While the standard deviation between the categories in each metric diminished, the most populous category in each metric in the pool of 70 candidates remained the most populous category in the group of 30 participants. The only exception was gender, which was equalised in the final selection.

For our record purposes, each participant was requested to sign a copy of the study participation informed consent form, mentioned above. We accepted electronic signatures on the form, or scans or photographs of signed hard copies of the consent form.

Our attrition rate was low: one participant withdrew from the study shortly before it commenced and was not replaced. The demographic composition of the group of 29 participants is presented in **Table 1**. It should further be noted that because of electrical power failures, one participant could not attend the first day of deliberations and another missed the last day of deliberations. The 29 participants were each paid R1800 as compensation for their time.

**Table 1:** Demographic composition of the participants.

Variable	Categories	n (%)
Gender	Male	15 (51.7)
	Female	14 (48.3)
Race group	Black	15 (51.7)
	Coloured	2 (6.9)
	Indian	5 (17.2)
	White	7 (24.1)
Religion or belief system	African traditional	4 (13.8)
	Agnostic	3 (10.3)
	Atheist	3 (10.3)
	Atheist/Humanist	2 (6.9)
	Christian	10 (34.5)
	Hindu	3 (10.3)
	Jewish	2 (6.9)
	Other	2 (6.9)
Education	High school	11 (37.9)
	Diploma	6 (20.7)
	Bachelor’s degree	4 (13.8)
	Honours degree	3 (10.3)
	Master’s degree	4 (13.8)
	Other	1 (3.4)
Age	18–29	14 (48.3)
	30–39	8 (27.6)
	40–49	4 (13.8)
	50–59	3 (10.3)

### ii. Measures

The study was structured around three themes: HHGE for (1) preventing the inheritance of genetic health conditions, (2) immunity against developing health conditions, and (3) the enhancement of genetic traits. Each theme contained four or five policy proposals. These policy proposals were:

#### Theme 1: preventing inheritance

Provided that it is safe and effective, our country’s laws should allow parents to choose to use genome editing before a child’s birth to—

P1. prevent the child from being born with a serious heritable disease like sickle cell anaemia, muscular dystrophy, or Alzheimer’s.
P2. prevent the child from being born with a less serious heritable disease like asthma or eczema.
P3. prevent the child from being born with a disability like deafness or blindness.
P4. prevent the child from being born with albinism.
P5. prevent the child from being born with Down’s syndrome.

#### Theme 2: Immunity

Provided that it is safe and effective, our country’s laws should allow parents to choose to use genome editing before a child’s birth to—

P6. make the child immune to contracting a serious disease like TB during the child’s life.
P7. make the child immune to contracting a serious but mostly preventable disease like HIV/Aids during the child’s life.
P8. make the child immune to contracting Covid-19 during the child’s life.
P9. make the child immune to contracting an illness like the flu or a common cold during the child’s life.

#### Theme 3: Enhancement

Provided that it is safe and effective, our country’s laws should allow parents to choose to use genome editing before a child’s birth to—

P10. influence talents such as how intelligent or athletic the child will be.
P11. influence personality traits, such as how aggressive or cooperative the child will be.
P12. influence the sexual orientation of the child.
P13. determine aesthetic characteristics, such as the child’s skin tone (to be lighter or darker) or the child’s eye colour.

In the case of each proposal, participants had three possible responses:

- Yes, always.
- Yes, subject to certain conditions.
- No, never.

The main amendments to our original protocol based on the pilot study were: (a) Whereas safety and efficacy were originally mentioned as an example of a condition for allowing a policy proposal, given the ubiquitous nature of safety and efficacy as a condition, we made it a general assumption built-in to the policy proposals. (b) Whereas the original protocol required feedback per policy proposal on who should be responsible to pay in the event that the proposal was allowed, this aspect was removed because of time constraints. It also allowed the discussion to focus more on the core issue of whether a certain use of HHGE should be allowed. (c) In the original protocol, TB and HIV/Aids were combined in one proposal. However, during the pilot study we found our pilot participants viewed these two diseases differently. Based on this observation and because these diseases are epidemics in South Africa, we decided to treat them separately. (d) In the interest of time, we combined athleticism and intelligence in a single ‘talent’ proposal, and cooperativeness and aggression in a single ‘personality traits’ proposal.

### iii. Methods

The DPE took place via Zoom over three consecutive weekday evenings. We offered to purchase internet data for the participants to ensure that all participants could fully participate throughout the three evenings of deliberations. Twenty-four participants used this offer. All participants and facilitators had their videos on at all times to make the online experience more personal and to allow the facilitators and participants to pick up on non-verbal cues. In terms of internet connectivity, the online deliberations generally proceeded without problems or interruptions. As mentioned above, one participant could not attend the first evening and another missed the third evening of deliberations, both because of power failures.

The first evening’s engagement, which included introductions, lasted about three hours. The second and third evenings’ engagements were each two and a half hours long. All deliberations were recorded and transcribed (S1 File). Each evening’s engagement focused on a theme and was structured as follows:

1. *Welcome*. The plenary facilitator welcomed everyone and introduced the evening’s theme and policy proposals.
2. *Breakaway session 1*. The participants were randomly divided into six breakaway groups of five participants per group, each with a breakaway group facilitator. The first breakaway session was timed to be exactly 25 minutes in duration.
3. *Plenary session 1*. The participants returned to the plenary meeting and the plenary facilitator asked a randomly selected member of each breakaway group to report on the group’s deliberations. Participants generally were also invited to supplement the selected rapporteurs’ reports. To encourage consensus building, the plenary facilitator would summarise the main issues raised.
4. *Breakaway session 2*. The participants were again randomly divided into six breakaway groups of five participants per group, each with a breakaway group facilitator. The second breakaway session was timed to be exactly 35 minutes in duration.
5. *Plenary session 2*. The participants returned to the plenary meeting and the plenary facilitator asked a randomly selected member of each breakaway group to report on the group’s deliberations. Participants generally were also invited to supplement the selected rapporteurs’ reports.
6. *Voting*. The evening concluded with a Zoom poll on the policy proposals of the evening’s theme. After all participants submitted their votes, the plenary facilitator shared the results onscreen with all participants before concluding the meeting.

During the welcoming at the first evening’s meeting, the plenary facilitator reminded the participants that they should make reasonable attempts to find consensus, but that consensus would not be forced on anyone. Facilitators remained neutral on the issues and endeavoured to (a) provide fair opportunity for all participants to contribute their opinions, and (b) encourage participants to engage with each other’s opinions and reasons, so as to explore potential areas of consensus. The facilitators received training by a local mediation and facilitation expert, which included training in using various techniques to accomplish (a) and (b) above. The facilitators had the opportunity to practise their facilitation skills during the pilot study. The trainer observed the pilot study and gave individual feedback to each facilitator after each of the three sessions of the pilot study. Note that although the deliberations were facilitated in a consensus-seeking fashion, consensus was not forced, and reaching actual consensus was not a criterion for the success of the DPE study. Instead, the clear articulation and documentation of all positions were the success criteria.

A genetics expert was also present to answer any technical question related to genome editing. She was only called on once during all the plenary sessions to clarify a technical question. We suggest that the low level of technical uncertainty vindicates the entrance exam approach.

Voting on the 13 policy proposals also took place via online polls a week before and finally a week after the deliberations. In other words, the participants’ opinions were captured at three times: A week before the deliberations (T1), at the end of every evening of deliberations (T2), and a week after the deliberations (T3). The voting data are recorded in S1 Data.

The deliberations during the plenary sessions of the three evenings were recorded, transcribed and pseudonymised–see S1 File. Our analysis of the content of the deliberations is based on this transcript of over 24 000 words.

## III. Results

**Fig 1:**
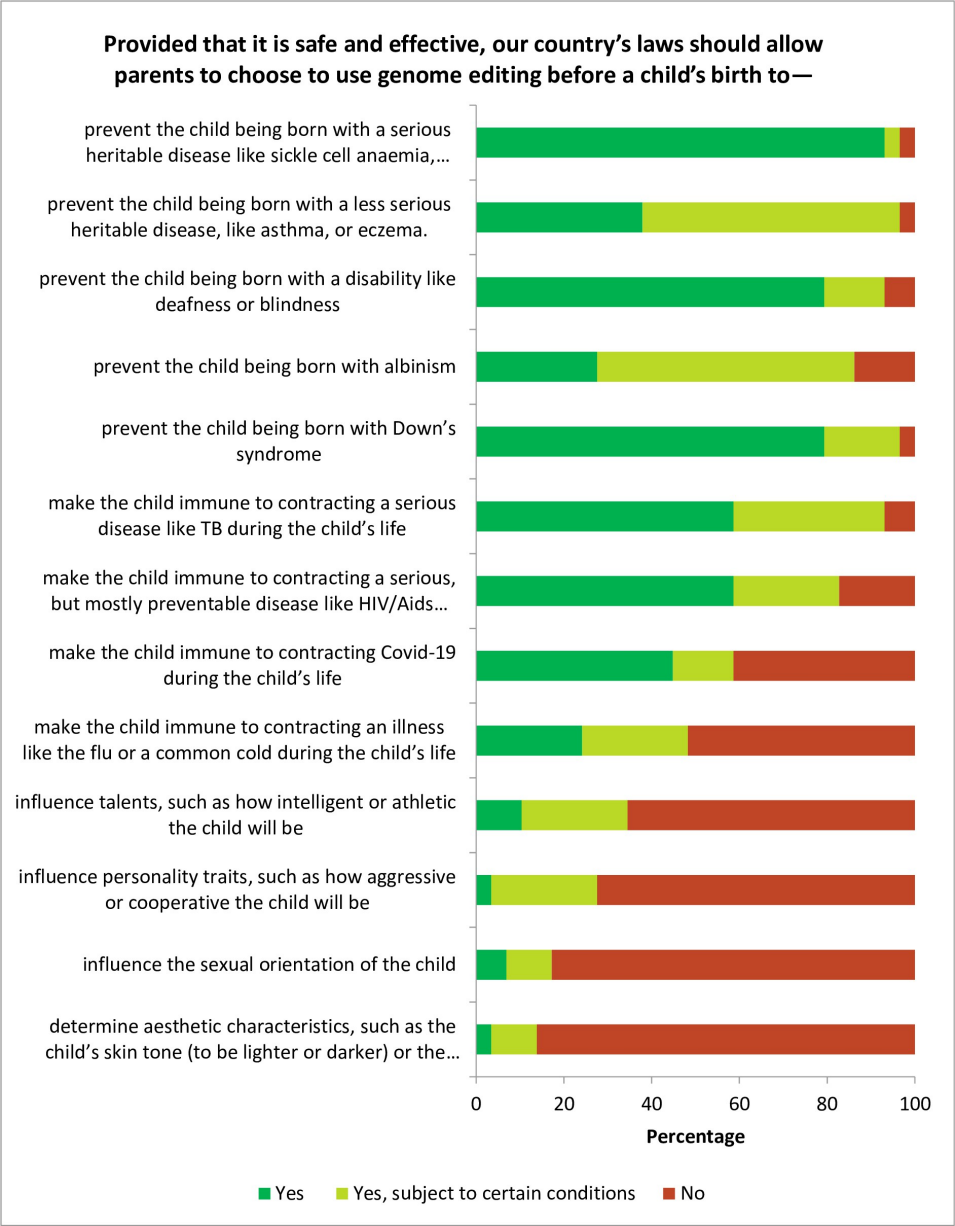
Voting results a week after deliberation.

The voting results at T3 are presented in **Fig 1**. There was overwhelming support for allowing the use of HHGE to prevent genetic health conditions, although a majority felt that this should be subject to certain conditions in the case of less serious heritable diseases generally, and in the case of albinism particularly. Allowing editing for immunity enjoyed overwhelming support in the case of TB and HIV/Aids, but had weaker support in the case of Covid-19. The turning point in the support of allowing HHGE was editing for immunity against illnesses such as flu and the common cold, which was opposed by a small majority. Significant majorities opposed allowing HHGE for enhancement.

The dominant paradigm during the deliberations was balancing health benefits (and associated improvements in quality of life) with unforeseen health risks such as loss of natural immunity. While the primary gist of this health-focused risk-benefit paradigm was focused on the individual, it was often intermingled with references to public health (such as costs or cost savings for the public health system). The seriousness of a health condition emerged as the determining factor for the policy choice of whether to allow an application of HHGE. The preventability of a disease through conventional means was debated, but had a minor impact.

Equal access to HHGE *qua* healthcare service emerged as an important value. This affected considerations of costs to the healthcare system, which in turn underscored arguments that HHGE should only be allowed in the case of serious health conditions.

Going beyond the parameters of the policy proposals, participants suggested that the South African government should do more to support research into HHGE by South African scientists in order for South Africa to establish itself as a leader in this new technology, rather than just being an adapter.

In the subsections on each theme below, we discuss the results of each theme in more detail. The Stuart Maxwell test (generalised McNemar test) was used to determine if there was a significant change of opinion as a result of the deliberation. This is an appropriate method to do analysis on paired ratings across two points in time because the Stuart Maxwell test is applicable for >2 rating categories. Note that we are not trying to project results onto a population.

### i. Theme 1: Prevention of genetic health conditions

Significant changes in opinion in favour of allowing the *unconditional* use of HHGE occurred from T1 to T3 with P1 (prevent the child being born with a serious heritable disease, from 65% to 93%, p = .033) and P5 (prevent the child being born with Down’s syndrome, from 45% to 79%, p = .018). This trend was also present but less pronounced with P2 (prevent the child being born with a less serious heritable disease, from 24% to 38%, p = .18), P3 (prevent the child being born with a disability like deafness or blindness, from 66% to 79%, p = .405), and P4 (prevent the child being born with albinism, from 21% to 28% p = .405).

Despite the general move of opinion towards the *unconditional* use of HHGE in this theme, in the case of two policy proposals, P2 (prevent the child being born with a less serious heritable disease) and P4 (prevent the child being born with albinism), the majority of participants favoured the *conditional* use of HHGE. This raises the question: What were the conditions that these participants had in mind?

In the case of P2 (prevent the child being born with a less serious heritable disease), participants viewed the examples of less serious heritable diseases, namely eczema and asthma, as sufficiently serious to justify using HHGE, but subject to the condition that more serious health conditions such as the examples provided in P1 be prioritised in the allocation of healthcare resources. This speaks to the value of equal access to HHGE *qua* healthcare service, contoured by the reality of limited resources.

Social stigma associated with disabilities—especially albinism—was frequently discussed. The issue of social stigma was perceived as ambivalent, as it could be an argument *for* the use of HHGE (by preventing the prospective child from being an object of social stigma) and *against* (by potentially reinforcing existing social stigma). Majority opinion converged on the position that social stigma has causes independent of HHGE, and that HHGE should not be used as a proxy to address these causes. Because of this, most participants did not feel that resolving social stigma should be a precondition for the use of HHGE. In the case of P4 (prevent the child being born with albinism), a majority supported allowing the use of HHGE to prevent albinism subject to the condition that it should go hand-in-hand with educational campaigns to de-stigmatise albinism.

### ii. Theme 2: Editing for immunity

Similar to the use of HHGE for the prevention of genetic health conditions discussed above, support for allowing the *unconditional* use of HHGE increased significantly from T1 to T3 with P6 (make the child immune to contracting a serious disease like TB, from 38 to 59%, p = .019). A less pronounced impact was found for P7 (make the child immune to contracting a serious, but mostly preventable disease like HIV/Aids, from 41 to 59%, p = .127). However, the opposite occurred with P8 (make the child immune to contracting Covid-19), where *opposition* to allowing the use of HHGE increased from 24 to 41%, p = .201. A lesser majority of 59% of participants remained in favour of either the conditional or unconditional use of HHGE for immunity against contracting Covid-19. The support for allowing HHGE ends with P9 (make the child immune to contracting an illness like the flu or a common cold), where a small majority voted in opposition at T3. P9 experienced slight increases from T1 to T3 at the poles of allowing the unconditional use of HHGE (from 14 to 24%) and not allowing the use of HHGE (from 48 to 52%, p = .782).

Given the dominant health-focused risk-benefit paradigm, HIV/Aids, TB, and, to a lesser degree Covid-19, were perceived as sufficiently serious diseases that justify the use of HHGE, while illnesses such as the flu or a common cold were not. Of note was that the massive economic cost of HIV/Aids, TB and Covid-19 in South Africa was repeatedly mentioned during the deliberations. It was pointed out that if HHGE offers a solution to make persons immune to these diseases, it would not only amount to a significant health benefit but also to a long-term cost saving for the South African public health system.

### iii. Theme 3: Editing for enhancement

Noteworthy majorities opposed allowing HHGE for enhancement. (As mentioned above, we use the term ‘enhancement’ to refer to non-health-related purposes.) There were no significant changes in opinion from T1 to T3 with any of the policy proposals. Given that HHGE for enhancement offers no health benefit and given the ascendancy of the health-focused risk-benefit paradigm, allowing HHGE for enhancement had limited support. However, during theme 3, a new paradigm emerged in parallel to the dominant health-focused one: weighing the procreative autonomy of prospective parents against (a) the value of diversity at society level, and (b) the autonomy of prospective children at an individual level. In respect of (a) it was argued that prospective parents may be susceptible to following social trends, which could lead to the loss of human diversity; in respect of (b) it was argued that prospective parents should not seek to influence certain characteristics of their prospective children, as these characteristics properly fall within children’s domain of autonomy.

Intelligence made for an interesting case, as it was argued that HHGE for higher intelligence presents a special case of an enhancement that would not only benefit the genome-edited individual, but also benefit society at large. In response, several concerns were raised, inter alia that this could set in motion the self-directed evolution toward a new kind of human that may find it difficult to identify with un-edited humans.

## IV. Discussion

### i. General

This study shows that well informed, considered public opinion in South Africa supports allowing HHGE for health-related purposes if the relevant health condition is deemed sufficiently serious. This finding is aligned with previous research that shows that approval of applications of HHGE for health-related reasons correlates with the seriousness of disease [22]. This appears to be motivated by the participants’ opinion that using HHGE is acceptable if it aims to improve the quality of life of prospective children, which is something that has also been a prevalent theme in preceding research [32].

However, it should be noted that the question of whether a particular health condition is deemed sufficiently serious is context-specific and may therefore differ between different countries. This is evident from the fact that South African participants took the threat of infectious diseases such as TB and HIV very seriously, unlike participants in other countries who have described such applications as immoral [33], or as the beginnings of a ‘slippery slope’ towards perceived unethical enhancements [20]. We explore why this is the case in more detail below. In cases such as these, where public engagement results differ from results found elsewhere in important ways, the value of the DPE methodology adopted in this study is most evident. With access to the deliberations that occurred between participants, we have insight into the reasoning that informed participants’ decision-making. This would otherwise have been absent had participants merely been polled. For instance, in relation to the study participants’ openness to HHGE being used for HIV and TB, it is evident from the fact that the economic cost associated with these diseases was a major talking point (as we allude to above), and that the weighing of long-term economic costs was a significant contributor to participants’ views that HHGE may be used to prevent them.

The study also adds to the studies in other countries that showed that the difference between the use of HHGE for prevention (viewed as the ‘correction’ of a genetic ‘error’) and for immunity (viewed as ‘enhancing’ the ‘normal’ genome to make the individual immune) did not have significance ethical relevance from the public’s perspective [24, 25]. What mattered to the participants was the unifying element of the purpose or intended outcome of the HHGE application. Where the purpose was promoting the health of the prospective child, applications were viewed favourably regardless of whether they could be viewed as ‘enhancements’. It is interesting to note that in light of this, while the participants referred to the potential dangers of HHGE for future generations—such as: (a) the loss of human diversity; (b) the creation of genetically modified trans-humans who can no longer relate to un-edited humans; and (c) genetically modified persons being victims of suffering due to social stigma associated with HHGE—in most cases they did not feel that these dangers were sufficiently significant to justify not allowing HHGE. Ostensibly, participants reached their conclusions by weighing the dangers they foresaw for future generations against the value of the purpose served by the HHGE application being discussed.

### ii. Country-specific context is important

To illustrate the importance of country-specific context, we focus on the issue of the preventability of certain diseases using conventional methods—in other words, methods other than HHGE. Given the health-focused risk-benefit paradigm, the preventability of a disease using conventional methods means that the same health benefit can be attained without the potentially unknown health risks of HHGE. This erodes the justification for allowing HHGE for immunity against a disease with a high level of preventability using conventional methods. In this light, the study required participants to consider all three of the current epidemics in South Africa: TB, HIV/Aids, and Covid-19.

TB is the foremost cause of death in South Africa [34]. All children in South Africa receive a TB vaccine at birth; however, this vaccine (BCG) is only effective against certain severe forms of TB in infancy (such as TB meningitis) and not against pulmonary TB. TB patients are often not quarantined during the diagnostic process, and can therefore expose the community at large to infection [35]. In addition, there are practical challenges—a lack of TB quarantine facilities and non-compliance with isolation procedures in hospitals means that the quarantine of TB patients is often not achieved [36]. Furthermore, TB patients often do not adhere to treatment regimens, leading to the development of multi-drug-resistant TB [37, 38].

While TB is spread through particles in the air when a sick person sneezes or coughs, HIV can only be contracted thought unprotected sex and contact with blood. However, despite the apparently easy ways to prevent the spread of HIV/Aids—and massive awareness campaigns by government and civil society—it has remained an epidemic in South Africa. Also, there is no vaccine for HIV/Aids.

Covid-19 presents a complex situation. At the time that the deliberations took place, only 28% of South African adults have been fully vaccinated [39]. Furthermore, it is estimated that 29.2% of the adult South African population may be described as ‘vaccine hesitant’ [40]. While this hesitancy is partly rooted in a general distrust of government, it is also rooted in a belief that vaccines are ineffective [41].

In light of these country-specific experiences and the problems with the preventability of the three epidemics in practice, it could have been expected that preventability would be discounted as a decision-making factor. This was especially true in the case of TB and HIV/Aids, but also with Covid-19: 7% of participants oppose the use of HHGE for immunity against TB, and slightly more, 17%, oppose the use of HHGE for immunity against HIV/Aids. Although the use of HHGE for immunity against Covid-19 was eventually opposed by 41% of the participants, a majority (59%) still supported its use against Covid-19. In countries where TB and HIV/Aids are not epidemics, and where Covid-19 vaccine hesitancy is less than in South Africa, these results may well be expected to be different.

These findings give credence to assertions by scholars that the ‘therapy’ versus ‘enhancement’ binary is a problematic baseline for governance [14], especially in the African context [29]. This binary evidently fails to capture what the public seems to consider morally relevant: the purpose of an intervention. There are clearly contexts within which applications of HHGE that may be classed as ‘non-therapeutic’ or ‘enhancement’—according to some broader definitions thereof—would be acceptable to the public. This suggests that policymakers should not make determinations about acceptable uses of HHGE technologies based on where they fall in the therapy-enhancement binary, but rather based on case-by-case, context-specific evaluations.

### iii. Limitations

As acknowledged in our study protocol, the online format has limitations—especially in a country such as South Africa where internet access is not ubiquitous. Despite this limitation, we were able to involve a diverse range of participants, as shown in **Table 1** above. Twenty-four participants took up our offer to buy them internet data, which eliminated this potential financial obstacle to participation.

A drawback of the online format is that the environments of the participants cannot be controlled. This had two main effects: First, a small number of participants were less interactive than the others and sometimes even appeared distracted. However, the vast majority of participants was highly involved in the deliberations. Second, because of electricity power failures that have become common in South Africa, one participant could not join the first evening of deliberations, and another participant could not join the last evening of deliberations. We suggest that this attrition is relatively small and did not have a significant impact on the study.

On the positive side, the online format has proven to offer distinct benefits, such as national reach and time and cost savings. On the issue of national reach, although this was not a selection criterion, it should be noted that we had participants from six of South Africa’s nine provinces.

Our methodology of structuring the deliberations into three general themes may also be a limitation. Although the facilitators asked the participants to address each of the policy proposals, the deliberations often evolved into arguments of a more general nature pertaining to the theme. Given the similarities between the policy proposals belonging to a theme, this is not always problematic but might have led to faulty generalisations in the arguments.

In the context of *conditionally* allowing HHGE, it was not always clear whether participants contemplated conditions *qua* (a) policies that must be in place before allowing a particular use of HHGE, or (b) on-the-ground realities that must first be achieved before allowing a particular use of HHGE. Consider, for example, access to HHGE as a condition. Does it mean that a particular use of HHGE may only be allowed if (a) government has policies in place to promote access to HHGE, or (b) the on-the-ground reality is that people have access to HHGE? Although (a) and (b) can and ideally should converge, it is not always the case in practice, and it is therefore something that could be better clarified in future studies.

We acknowledge that the online deliberative engagement format means the exclusion of views from certain groups of persons, including those who do not have access to electronic devices, as well as persons who are unwilling to participate in heterogenous discussion. That being said, we believe that by ensuring a wide array of diversity in our participant group, we have taken sufficient measures to ensure that viewpoints from various South African backgrounds had an opportunity to be represented. We do not believe that the exclusion of the persons mentioned above influenced the discussions to any significant degree.

## V. Conclusion: A call for policy development

In this paper, we have shown how DPE as a methodology can be effectively implemented using an online platform, and how this approach has the advantage of providing insight into the well-informed, considered views of the public. The focus on deliberation and consensus-forming caused participants to engage critically with deeply held views and values, and in some cases they even changed their initial position. This can be of value to the development of governance structures for novel technologies such as HHGE, as policies can be based on the well-informed, considered views of the public, rather than knee-jerk reactions which may be adversely affected by a lack of knowledge, or by a ‘yuck’ response to a novel technology solely because it is new [42].

So what do the results of this study mean for South Africa?

The governance of HHGE in South Africa is disjunct. While South African law can be interpreted as allowing a pathway for research on HHGE, the legal regulation of the clinical application of HHGE is uncertain [15]. However, the leading ethics guidelines in South Africa prohibit all research on, and the clinical application of, HHGE [15]. Thaldar et al [15] propose five principles—aligned with the values of the South Africa constitution—to guide policy development for HHGE in South Africa:

- Principle 1: HHGE should be regulated, not banned.
- Principle 2: Use the well established standard of safety and efficacy.
- Principle 3: Non-therapeutic uses of HHGE may be permissible.
- Principle 4: Respect parents’ reproductive autonomy.
- Principle 5: Promote the achievement of equality of access.

The results of this study affirm the five principles proposed by Thaldar et al [15] and provide them with more detailed texture. The study shows that well informed participants, after deliberation with their peers and after time for self-reflection, overwhelmingly support allowing prospective parents to use HHGE for preventing genetic health conditions and for immunity against serious diseases—even diseases that are mostly preventable—provided that such uses of HHGE are proven to be safe and effective (Principles 1, 2, 3 and 4). During deliberations, it was uncontested that government should allocate resources to promote equality of access to (permitted) HHGE healthcare services (Principle 5). Moreover, it was uncontested that government should allocate resources to promote scientific research into HHGE.

Accordingly, there is not only a legal–ethical case as presented by Thaldar et al for not obstructing research into and the eventual clinical trials of HHGE in South Africa, but also a political case based on well informed, considered public opinion. This calls for urgent revision of South African ethics guidelines that currently prohibit research on HHGE, and for dedicated HHGE legal regulations that provide a clear and comprehensive legal pathway for researchers who intend to conduct HHGE research and clinical trials. Also, South African public research funding agencies should include HHGE in their funding mandates.

## Supporting information

S1 File Transcript of all three evenings’ plenary deliberations. (DOCX)

S1 Data Voting by participants on each policy proposal at T1, T2 and T3. (XLSX)
