# Supplementary material for: A deliberative public engagement study on heritable human genome editing among South Africans: Study results
Source: PLoS One. 2022 Nov 28;17(11):e0275372. doi: 10.1371/journal.pone.0275372 (PMC9704621; doi:10.1371/journal.pone.0275372)
Supplement: S1 File — (DOCX) [file pone.0275372.s001.docx]

# A deliberative public engagement study on heritable human genome editing among South Africans: Study results

Donrich Thaldar, Bonginkosi Shozi, Michaela Steytler, Gill Hendry, Marietjie Botes, Ntokozo Mnyandu, Meshandren Naidoo, Siddharthiya Pillay, Magda Slabbert, Beverley Townsend

--

**S1 Document.** Transcript of all three evenings’ plenary deliberations

**Day 1**

**First plenary**

Donrich: There we go, ok! Let’s mute, let’s mute everybody. Great stuff. Welcome everybody! Well, I must say that my group, in my breakout [session], had a very productive session, we were actually finished with our discussions a few minutes ahead of time. So, now it’s time for people to report back, and I can't wait to hear about your progress. So, first, Bongi’s group, if I may ask Participant 14 to report back, please.

Participant 14: Alright, good evening, guys. I hope if I miss something my group members will chip in and add where I missed on certain points. We couldn’t manage to answer questions 4 and 5, but on the first question there was a consensus and we agreed that the government should certainly allow parents to choose to prevent the child from being born with serious heritable diseases like sickle cell. Participant 7 raised the point of, where she had a friend, from experience she knew that sickle cell is quite, very painful, quite painful, and she has seen how it has affected people close to her and she was of the opinion that there should be no conditions to this. Participant 24 as well agreed, if people were to die young, certainly this should be prevented, and Participant 18 as well agreed. Participant 23 agreed [subject] to certain conditions, but ultimately, we had a consensus that we agree but I think the condition is that the technology is widely accessible to everyone and then on the second question, which was preventing the child from being born with asthma, there was a consensus and then, was it asthma? No, born with, yah, born with asthma. We had a consensus, but a little bit of disagreement in terms of, we agreed, but subject to certain conditions. Someone else particularly, I think it was Participant 18, mentioned that asthma and eczema could be treated and probably this technology shouldn’t be that, shouldn’t be used, you know. But Participant 7 chipped in and mentioned how, sometimes, in as much as it’s treatable, one of, two of her friends passed on because they could not access their pump, asthma pump. I think, I also agree to the technology being used but subject to certain conditions. On the third one, on the issue of blindness and deafness, there was a question around whether blindness and deafness was heritable, but it was answered, yes it was. So, I think there was total agreement, though we got cut off, but overall, I would say there was, we reached a consensus, but our conditions were quite different, I mean, it revolved around the seriousness of illness, and the issue of patients making, or parents, making an informed decision, being informed about future implications or the side effects of such a treatment before they could agree to it.

Donrich: That’s an interesting point that you’re raising, namely the informed decision making by parents.

Participant 14: Absolutely, I think it was Participant 24 who raised it, or was it Participant 23? I think it was Participant 23. So yeah, essentially there were different conditions raised from my fellow group members and…

Donrich: Can you quickly tell us what the conditions were?

Participant 14: One of them was that the seriousness of the condition should be one of the limiting factors in terms of the accessibility of the technology. Accessibility of the technology to everyone, the pricing of the technology, some issues of how genetic therapy, I know it’s not within this context, but genetic therapy seems to be quite expensive in the US, treatment of blindness is more like $850 thousand in the US, SMA [spinal muscular atrophy] $1.25 million. So the regulation of policies around these pharmaceutical companies, you know, prices should be fairly governed and the government as well should make this quite accessible. But also owing to the fact that this would be done at fertility clinics, this implies this would be within the private sector and that also would also bring in the factor of regulation around that. But also, the government should play a huge role in being quite competitive and in being part of this new change and making this quite accessible to everyone in order to, not to divide the gap further to increase the inequalities within our societies as it is already quite an inequitable society.

Donrich: Yah. So in summary, basically three issues there, namely the seriousness of the condition, access, broad public access, and governance of prices which I think overlaps with that one. Participant 14, I would like to go onto the other groups, are there any other important aspects that you would still like to highlight or are you happy?

Participant 14: I’m happy.

Donrich: Ok, perfect. We’ll then do my group, and if I may ask Participant 11 to do the report please.

Participant 11: In my group we actually reached consensus on all the five, but then there was a problem with albinism because at the beginning I think Participant 22 and Participant 28 didn’t actually agree with all of us because they didn’t look at it in a way of like albinism affecting people health wise, and then after raising the issue of people living with albinism having higher chances of skin cancer, they actually were able to see it from a health point of view and then they were able to agree that parents should be given the option to genetically edit their embryos in order to prevent their kids being born with albinism. And then, with all of them, we all agreed on gene editing.

Donrich: So, agreed on all five acceptable. Thank you very much Participant 11. Next the third group was Marietjie’s group. If I may ask Participant 2 to report please.

Participant 2: We only got to question 3, we were deliberating a lot, so for the first one we all agreed that for diseases, major diseases like that, we would do gene editing. On the second one, we were quite busy for a while. We agreed, but with certain conditions. Things that, or diseases and stuff that influences your way of life. If you can treat it with medicine to not do it [gene editing], but with asthma, that’s life threatening, then gene editing would be beneficial. On the third one, for disabilities, also, depending on what type of disability. For not that major disabilities like for ears or toes or stuff like that, then it doesn’t affect the quality of life, then it’s not needed. But deafness, blindness, those type of things, then definitely gene editing. And yah, that’s all, thank you guys.

Donrich: Thank you, Participant 2. You mentioned something, namely that, if the condition can be treated with medicine, then it influenced your thinking. Why would that influence your thinking?

Participant 2: Like, eczema, it’s an annoying thing, but it’s treatable. Participant 16 and Participant 5 also mentioned it. Asthma, that can be a very difficult thing to deal with, even though you do get treatments for it, but if you don’t have the money for it, then you can’t really live a long life and it can be very dangerous. With some other things, you can live with it, it’s not going to affect your life that much.

Donrich: Thank you Participant 2, I see that Participant 19 has her hand raised, but Participant 19, can I ask you, I'm going to give you an opportunity after we’ve gone through all the groups. I just want everything on the table and then I’ll give you an opportunity. So, over to Magda’s group, Participant 1, if you can report for us please.

Participant 1: On question 1, we were all in consensus, it was yes. I think on question 2, we were three yeses and two with certain conditions. The one thing that I can remember, one of the certain conditions was if it will be accessible to the poor, the rich and the poor, because you can, if you are, obviously if you have more money you can treat it or if you are richer then you will be able to access the gene editing for asthma and eczema and the poorer person will not be able to access it so there should be fairness in that. On question number 3, was, I can’t remember now, yah, but, the albinism we were also not in consensus but we had I think three or four yeses and then in there was said with certain conditions that people should actually be educated regarding albinism and we should try and be live in a better world but from the health side then it should be yes. With the Down’s syndrome we were also yes and then with certain conditions. I think with the Down’s syndrome there was mentioned toward parenting and that you should also because people felt at least I felt that it’s about the child’s future after the parent passes away but there was a feeling that you should actually make financial arrangements for your child after you pass away and then for the deafness and the blindness, I think we also had yeses and also some with certain conditions because there was felt that ASL is available and that there is braille available for blind people.

Donrich: Thank you, Participant 1, also very insightful. I will move onto Meshandren’s group. If I may ask Participant 4 to report please.

Participant 4: Oh ok, question 1, for serious heritable diseases, all agreed, yes, it should be used. Then question 2, less serious heritable diseases, we also agreed but with certain conditions. Conditions being: if it is treatable then, you know, but then there was also, it was my question, when a person is living in a very high temperature place, where the sun is 30 degrees up, what can that person do because a person who has eczema, cannot, it is hard for him or her to live in that certain place with high temperatures. Then, we agreed on disability, we said yes. And then, it came to Down’s Syndrome. Ok let me go to, we also agreed certain to, certain conditions. But the debate was on albinism, specifically, Participant 25 said that gene editing can be done if the parent feels like the person, or the child living with albinism, is a life threatening issue. Ok, and so I questioned, it was my question, there are certain parts in KZN where a person, when a person is living with albinism, is regarded as the key ingredient for a herbal medicine, therefore they are killed for being, for living with albinism, so I thought, or asked, isn’t that life threatening? Wouldn’t you want to spare your child that misery? Because you might never know what will happen. As Participant 1 said, that there is a, when a parent passes on and then a child is left. So what will happen to your child living with albinism? So that was, we agreed, but certain [subject] to conditions.

Donrich: Thank you. Thank you very much Participant 4, and thank you for the example there from KwaZulu-Natal. Then, Ntokozo’s group, if I may ask Participant 6 to report please.

Participant 6: So, the general consensus for all of the questions was agree with certain conditions and the reason why, the main reasons were because of quality of life that is effected as well as the cost implications of all of these disabilities or diseases. When it came to the stipulations and the issues that were raised, the main one that came up was demonstrated safety of the techniques and what the downstream effect would be in a couple of years and if it should be allowed, if that hasn’t been shown to be safe in the long run and what the gene interactions would be.

Donrich: Yah, yah. Well, if I may, well I think that’s an important concern. For the purposes of our deliberation we can make the assumption that it is safe. Where there any other conditions that were mentioned by your group, Participant 6?

Participant 6: Um, not any that I can recall off the top of my head, I was just sticking to the ones that were mentioned in the question.

Donrich: Ok, so I know that Participant 19 would like, well, raised her hand a few minutes ago to comment. I just quickly want to ask, anybody from any of the groups who feel that their position was not reflected in this report back session? Was forgotten? Who would like to supplement any of the reports? [silence] Well, then, Participant 19, over to you. [silence] I hope that Participant 19 doesn’t have connection issues now. But, I think Participant 19 can also make her contribution during the break, during the next breakaway session. I will just quickly summarise what I have heard during our reports back. Thank you for all the brave people who reported back to plenary. There seems to be general consensus that these five applications of gene editing should be allowed. Some feel, some people feel there shouldn’t be any conditions and some people feel that there should be conditions, at least for some of these five and, but I didn’t hear that there was an opinion from anybody that it should never be allowed, that it should be stopped. And some of the candidates for conditions, that were mentioned, were using the seriousness of the condition, or whether it can be treated later in life through medicine, to consider that as a criterion, and we must give good consideration – why is that a criterion if you want to use that as a criterion? Then the other issue that was mentioned as a potential condition was access, that people should have sufficient access, that people generally, us South Africans generally, should have access to this technology and that government should also make sure that the pricing and the accessibility of this is reasonable. And there I would like all the breakaway groups to consider the following: The issue of access was mentioned ­– should that be a precondition for parents being allowed to use this, or is the issue of access something that the government should legislate or intervene in parallel with this technology? So just give a bit of thought regarding exactly how the issue of access should be used or should be applied. Ok, there are two hands, I'm quickly going to give you an opportunity. Participant 19 first and then Participant 25.

Participant 19: Ok, so I'm wanting to comment on the issue of, can you hear me?

Donrich: Yes, absolutely.

Participant 19: Oh ok. I'm wanting to comment on the issue of if it’s treatable, then parents should not be allowed to have the option. Now, if it’s a choice that a parent can make or if it’s a choice that a parent can be allowed to either be able to or not be able to, then it comes down to choice. And if we [inaudible] these treatable diseases, is it something that we would prefer? [inaudible] When something has treatment, there is a trial and error that is attached, loss of productivity when treatment fails, so it’s something, just, I'm trying to say that just because the treatment brings the person with that disease back up to be on the same footing as everybody else, I'm speaking from experience, as somebody who lives with [inaudible], it’s treatable, but there’s a lot of downtime. There's a lot of failed treatment, and if my parent was to [inaudible].

Donrich: Ooh golly, I think our reception is not very good. Participant 19, if you can hear me…

Participant 19: be allowed the option, and living with this treatable disease, [inaudible]. If it’s treatable, then parents maybe shouldn’t be allowed to choose to, you know, do that [gene edit], but to me it feels like, just because something is treatable doesn’t mean that you now lead a normal life. [inaudible] A second point, in the breakaway groups, I mentioned….[disturbance]

Donrich: I think, lets save the debate for the breakaway groups, else our plenary will never be able to finish. I’m sure in your next breakaway group there will be ample opportunity for you to share. Participant 25, if I can quickly ask you – if you want to further the debate, my suggestion would be let’s do that in the breakaway groups, but if there’s something else, a question for clarity or so?

Participant 25: It’s not necessarily a question, but it’s just a point which was not raised. So, in my group I had raised that with things like disabilities as well as albinism, the gene editing should not be used, and the reason is simply that I feel like, you know, it could have a negative impact on society’s ability to be more accepting to so many conditions and to people who are not perceived as normal in society so that’s just something I wanted to raise.

Donrich: I think that’s very important and thank you for raising it and I would like everybody, in your next breakaway groups, to specifically consider that. That is a challenge, and how are we going to deal with that? Thank you for raising that, Participant 25. So, I highlighted the other issues that were raised and I have indicated to you how I’d like the breakaway groups to consider that. Ntokozo also raised his hand and he communicated with me through chat, he indicated that I might just want to create a bit of clarity regarding the questions again, so let me share my screen, just for a moment, before we go on our breakaway groups. I trust everybody can see that. So, our theme for the evening is serious heritable conditions and whether gene editing should be used for that. I will just read it again, so that we can all make sure we’re on the same page. So, provided that it is safe and effective, that is our assumption, our country’s laws should allow parents to choose to use gene editing before a child’s birth to: and then five things. The first one is serious heritable diseases, like Alzheimer’s and so on. The second one is prevent the child being born with less serious heritable disease, like asthma or eczema. The third one, prevent the child being born with a disability like deafness, blindness. Fourth one, prevent the child being born with albinism. Fifth one, prevent the child being born with Down’s syndrome. So those are the five ones, and if everybody is ready, then we will go to our second breakaway groups and I hope you’re very productive and try to find common ground and please remember what I have highlighted, the issue that Participant 25 mentioned now, and how that should be handled, and then the other two issues that were mentioned in the context of conditions, namely seriousness of a condition, why should that be a criterion? Give that some thought. And then also the issue of access, should it be a condition or should it be a parallel issue or duty on government? So, Michaela, if you’re ready you can put us in our breakaway groups. Facilitators, please remember to record the breakaway sessions.

**Day 1**

**Second plenary**

Donrich: Ok, we’re all back here. Welcome everybody, we are back to our final plenary for this evening. I must say, my group had a very interesting and very productive session. So, without further ado, I’m going to ask for report back…

Michaela: Prof, if I might suggest for the first group to please ask Participant 4, Participant 22 or Participant 26 to report.

Donrich: Ok, I will ask Participant 22, please.

Participant 22: Ok. Yah, we actually reached quite a good consensus. I can’t a hundred percent remember what it was, [laughs]

Donrich: Do you want assistance?

Participant 22: So if either Participant 4 or Participant 26 could help me slightly there. We covered several issues, actually, because we moved onto other ones after that, such as whether the government should cover it. I do remember we all agreed that initially the private sector may have to cover the cost because it may be expensive but the government and the private sector should work hand in hand and definitely for those who can’t afford it, especially for serious issues, should have it covered for them.

Donrich: Mmm. Perhaps Participant 26, if you want to cut in, because Participant 22 says he would like to have some assistance regarding your consensus.

Participant 26: Yes, in adding to what he said, we also talked about, you know, the social implications of gene editing and whether, you know, it should be a precondition or not for allowing the use of gene editing and I think we agreed that it should be a concurrent discussion that happens because, you know, we’d be editing genes and all of that for the benefit, and whatever social implications arise out of that, you know, it should be a discussion that happens concurrently.

Donrich: It’s a concurrent discussion?

Participant 26: Yes, it should be a concurrent discussion, but those social implications should then not influence our decisions in terms of, you know, something that is potentially life threatening and [gene editing] has potential to improve the quality of life of people. So, on accessibility, yes, as Participant 22 said, private sector and government should work towards achieving, you know, accessibility for everyone. One of the things we also said was for more serious conditions, maybe because government might not have the technical know-how or the expertise, it might be expensive, then, you know, private sector and government, or, government should then subsidise people to access that service, but also, you know, with a focus initially on more serious conditions and then later on with conditions that there is currently medical treatment for, you know. And then, what we also, what we were just discussing lastly was upon whether or not, if we’ve proven, we’ve met all our preconditions and, say, gene editing is safe, it’s a hundred percent safe, we’ve dealt with the issues of social implications, accessibility, and all of that, what then becomes of a parent who refuses to have their child, you know, who say, there is a genetic predisposition to a certain condition and there’s a high likelihood that the parent would pass that onto their offspring, but then the parent refuses, are we then going to view it as negligence, perhaps, as we do with the issue of vaccines? If you don’t vaccinate your child, you are essentially dooming your child and we view that as an offense, I believe, in terms of the law. So, in cases where all, gene editing is safe, its accessible, all the preconditions are met, would then refusing to provide that for potentially somebody who might, of course we can’t know with a hundred percent certainty, of course, that someone is going to get, whatever, you know, but there is a high, there are correlates, you know. For example, there is a high correlation of that, that you’d get breast cancer and then you don’t, if you refuse gene editing for that and then you know, is that neglect? What then should be the appropriate response for that?

Donrich: That’s a very interesting question, and I’m very glad that you guys are thinking that far ahead. So, thank you for group one, I appreciate it. I was facilitating group two. May I ask Participant 14 to report back, please.

Participant 14: Oof, again! [laughs] Ok, I would ask my fellow group members to chip in where I did not clearly reflect what was discussed in the group. I think the first one to go was Participant 21. He was in favour of accessibility to anyone, provided, as long as, that it [gene editing] is safe. On the issue of the question that was asked by, I think, Participant 25, he [Participant 21] mentioned, he doesn’t think that kids should be burdened with the responsibility of carrying the, how do I put it? The weight of how the society perceives the disability, you know, and also mentioned that it is also, parents, it should also be up to the parents to make a decision. They should have the freedom to make such a decision, and as a matter of fact, parents are empowering their children. And then, Participant 7 raised her concerns around the technology in terms of the power that we have, the access to what she called the book of life. She mentioned how this has an implication of rewriting the book of life and also mentioned some of my fears in terms of, we are in cybernetic era, for a lack of a better word. More of, we are turning into cyborgs, the information is quite vast and easily accessible. She mentioned how one of the founders of, rather, the discoverers of this CRISPR-Cas9, I think Jennifer Doudna, some speech where she decided to hand over the technology, where she wished that the technology could be handed over to the World Health Organisation and the United Nations. She [Participant 7] mentioned that it shouldn’t just be given to the government. We also spoke of how IP could be used to incentivise our government in terms of developing a policy that…

Donrich: If I can quickly interrupt you there, just to make sure that, if I can just quickly interrupt you because I just want to make sure that everybody understands. Participant 14 is speaking about IP. That’s an acronym for intellectual property – things such as patents and trademarks and copyright and so on. And here comes a very interesting suggestion. So, the suggestion regarding IP.

Participant 14: South Africa should generally develop a policy that would, that’s more imaginative, rather than being reactionary. I mean, this technology has been developed over the past twelve years and now it’s here, you know. The government should be clear in terms of incentivising how, incentivising researchers to develop and learn more about genetics and genome, develop technologies around that particular area, as we all know that this is the hottest topic on scientific discussions, economical discussions. Participant 9 also raised his concerns, he mentioned that he came from a scientific background, he mentioned the three Rs, I think as far as I remember, that’s reduce, refine, replace before carrying out, which are the preconditions for science before carrying out any research. He mentioned that we should be able to replace, whatever it is that you are doing research on, be it animals, be it, if I’m quoting him correctly. And he mentioned the issue of reducing, and he highlighted how this is actually a good thing because it would be reducing the number of people who would be burdened with sicknesses such as, heritable sicknesses, such as blindness and so on and so forth. Also mentioned the issue of refining which has to deal with reducing pain on the subjects that you are doing research on. He was in agreement that the technology should be easily accessible, and I think, if I remember him correctly, universal access should be a precondition. And Participant 12…

Donrich: Yah, if I can quickly interrupt you, Participant 14, I don’t think it’s necessary to go through everybody’s contributions, if you can just in brief, what are the main points that the group reached consensus on? So, I think the last one remaining was regarding access.

Participant 14: Yup. The issue of access, yes, we reached the consensus that the technology should be accessible to everyone, and the government should certainly play a role in making sure that this is accessible as this will reduce the number of disability grants that government will constantly pop out each and every month.

Donrich: There was an important issue regarding access, what did the group decide? Should it be a precondition, or should it be concurrent, in parallel to permitting [gene editing]?

Participant 14: On that one, from my understanding there was a bit of confusion on my side because I was not quite understanding whether, what does it mean to say access should run parallel to… parallel to… [sigh]

Donrich: If I may, if you are unsure about that, can I ask Participant 9 to quickly come in there? I remember you were part of that discussion.

Participant 9: Which point is that? You said if…

Donrich: The issue of access that we discussed. In your observation, what was the group’s opinion? Should it be a precondition, or should it be concurrent to allowing gene editing for these five issues?

Participant 9: So, from what I understood from what everyone said, a couple points that came up to me was that first of all, is, well, I asked the question is that, will the government actually fund this particular thing for everyone for it to be accessible to everyone? We came to the conclusion that it’s most likely that the government would not fund it fully because technically they do not see it as being like a global pandemic, it’s not preventing someone from living a full life, but then we did say that the government should be able to then, incentivise, they should, first of all, incentivise scientists by providing funding for research into this so that we can be leaders in this field and not just receptionary [sic] to what everyone else brings, and then second of all, government then also would then be able to subsidise the treatment in order for it to be accessible to everyone else.

Donrich: Thank you, Participant 9. I think, let’s proceed to Marietjie’s group and if I may ask Participant 18 to report for us please.

Participant 18: As a group we actually decided that Participant 28’s going to be the one to report for us.

Donrich: Oh! So your group made a decision, that’s fine, I’m not going to argue. So, it will be Participant 28.

Participant 28: Yeah they decided to choose me for my sins that I don’t know!

Donrich: [laughing] You’re very welcome, Participant 28!

Participant 28: Yah, but in terms of accessibility, we do agree with the first group. We decided that it should be accessible to all, but those who can actually afford it should be allowed that space to pay for it themselves, if they want to go to the private sector, maybe they have a preference for better facilities, they should be able to do that. And then everyone else, that cannot afford it, maybe the government can do their social duties there. Participant 1 also mentioned that the government has a programme where they plan to help people that can’t have children, maybe like with one child, like an intra vires type of thing. Maybe this programme can also be linked to that.

Donrich: Ok.

Participant 28: That was in terms of access. And then in terms of societal attitudes, unfortunately we were of the opinion that gene editing is not actually here to solve those problems, you know, it cannot solve our biases and our prejudices. So, if gene editing can help us to have fifty disabled people instead of a hundred, it’s progress for society and we should go ahead with it and then have an avenue to sort all the other problems. And then in terms of seriousness, yah, we were of the opinion as well that maybe very serious conditions can have priorities and should be attended to, but we must just be careful of situations that we think are less serious, but can get very, worse as a person grows older or as time goes on, because there are situations like that, in the beginning they don’t seem to pose any threat but as time goes on or as a person gets older, then it becomes a real problem, they become open to some serious disadvantages in life, so it can be considered and be seen like that. I don’t know if maybe the other members want to add something? [silence] No, ok.

Donrich: Good! Participant 28, thank you very much, the designated reporter from group three. So, let’s go to group four, Magda’s group. And may I ask Participant 13 to report please. I just see that we are quickly running out of time so if I may ask our reporters to be as concise as possible, please.

Participant 13: Yes, as for accessibility, we suggested that if the procedure of treating a person [gene editing] is simple, then the treatment [gene editing] should be available to everyone and for most illnesses. If the treatment is simple, we have no idea how the treatment would work, is this an injection or is this [inaudible] is this similar to COVID vaccine injection, or is it more cumbersome? So we have no idea what’s the expense of this treatment and what are the, is it more expensive to treat a more serious case, or is it done on a privileged case?

Donrich: That we don’t know at this stage. [laughs]

Participant 13: So we would prefer, any kind of disadvantage to a child should be treated and if it works out, basically, if the treatment is very simple, then we should go ahead and the government should subsidise it for everybody. Thank you.

Donrich: Good. Thank you, Participant 13. Thank you very much. Let’s proceed to Meshandren’s group. If I may ask Participant 5 to report, please.

Participant 5: Can you hear me?

Donrich: Yes, Participant 5.

Participant 5: Ok, on the question 1 we reached consensus that serious diseases should be gene edited without conditions. On question 2, less seriousness, we also reached consensus and some said with conditions and without conditions. Disabilities, less serious and serious, should be gene edited. We had, we were a bit split there. And with albinism, I thought that is definitely a cosmetic procedure because there is nothing wrong with an albinism, with a guy that has albinism. And with the last question, there was consensus that it should be gene edited from the start. And in terms of the accessibility, it should be free and it’s very important what is the severity of the disease, that should be taken in consideration.

Donrich: Thank you very much Participant 5. Over to our last group, Ntokozo’s group. If I may ask Participant 10 to report please.

Participant 10: Sure, so, our group, we discussed initially accessibility, you know, how accessible this should be. There was some suggestions as to, you know, obviously everybody had the consensus that if a technology is to be available, it should be available to anybody who chooses it. There were some discussions on how that should be funded, whether it should be funded through the private sector or through government. Obviously, you know, we live in a country where government struggles to provide people with basic services like water and electricity, so we didn’t all agree but, you know, perhaps the government should be a part of securing funding for this kind of thing for people who cannot afford it. And then, also on the accessibility front, just more towards, in the beginning should it be concurrent or should be conditional, right? Should we continue developing this and make it available to those who can afford it, or should we, you know, decide not to develop it until such time as it can be made available to everybody?

Donrich: What was the decision?

Participant 10: I think there was more or less a consensus, apart from a few concerns, that it should be made available initially to those who can afford it, as with any other technology, right. And later on, you know, as it becomes more and more affordable, it can then be made available as it becomes feasible. And then around the question of social implications like, you know, consideration against, does that then mean that we are discriminating against those who have those diseases? And I think there was a consensus there that, you know, we’re not necessarily discriminating against people who have those diseases, we’re making advances with technology to improve the life of all humans and give everybody the opportunity to be on a fair playing field. Rather than to be born with a disease that was entirely preventable. So, yah, there was more or less a consensus there apart from a few concerns regarding how individuals may perceive that, but we did mention that, you know, everybody will, there will be people who see things a certain way versus not, a different way to the general public. It looks like Participant 19 wants to jump in quickly.

Donrich: Participant 10, thank you very much, very insightful from your group. Participant 19, please.

Participant 19: Sorry, we didn’t reach a consensus on the second one, we were split. We had three people saying that doing this would be showing empathy, understanding that people with these conditions undergo or go through life difficultly and one person felt that it’s trying to fix people and not being accepting and they thought that instead we should maybe invest more energy into trying to accommodate those people.

Participant 10: That’s correct.

Participant 19: The other three people felt that it, when we say that we should do this, we’re not saying that we should stop trying to accept people, we just trying to extend empathy and try to prevent the difficulty before it arises. And then on the first point we said that the, whether or not this should be allowed should not be dependent on whether it’s accessible or not. The access will come later, it should be allowed with or without accessibility and through social movements and through things becoming cheaper and cheaper with time, then access will be then addressed by those issues, the cheapness of it and the social movement. Sorry, I didn’t want to take…

Donrich: No, thank you very much. It’s good to supplement, you know, we’re all colleagues and we help and supplement each other and it’s good to make sure that all the views are definitely on the table. Participants, now for the highlight of the evening, I'm going to ask you to complete the polls. Please keep in mind, consider everything that you’ve discussed and consider everything that you’ve also now heard from the other groups and their ideas and what they’ve also discussed. So, I'm going to launch this poll and if you could then, can you just confirm that you can see it? Thank you. Only the participants please, not the facilitators [laughing].

[voting]

Donrich: Participant 25, you’re welcome to speak.

Participant 25: Ok, so I'm not sure, with the last question, I couldn’t click confirm, I'm not sure if that button was there but I was just exited from the thing, so I'm not sure if my response was recorded.

Donrich: We will make a note of that, and I see we currently have, in fact, 100%, well, everybody have responded. So, yours were therefore recovered, thank you very much. Ok, I'm going to, oh, now It says again 28 of 29, oh golly, what is happening? If you’re still busy van you just indicate to me? If anybody is still busy? Ok.

Participant 19: I was kicked out and came back, but I had already filled it in, I don’t know if that’s affecting anything.

Donrich: Ok. Let me share with you the results. Can you indicate to me, can you see it on your screen? Marietjie, was that, ok thank you, thank you for confirming. So, for the first question, 85% yes, always and 19% subject to certain conditions. It changes with the second question, as you can see there, where majority decided it must be subject to certain conditions. I see your hand Participant 19, just give me a second. So, number 3, you can see the results there in front of you. And number 4, there was some on no, with the majority on subject to conditions and then the last one, as you can see, overwhelmingly yes and then it tapers down to no. So, this is, I think, quite interesting for all of us involved. Participant 19, over to you.

Participant 19: It says that I did not answer the questions when you shared, it says that you did not answer the questions. So, it’s possible that when it kicked me out it erased my poll answers.

Donrich: Ok, what I will do, because we are recording everybody’s answers, let’s me just close this. Michaela, you can advise me, but I think what we can do is afterwards just to make sure with I think Participant 25 indicated that there might be a problem, so we can just contact them tomorrow morning, and the same also with Participant 19. Michaela will contact you either tonight or tomorrow morning and then we will just separately record that and include that in our records. If anybody else is also concerned, well, Michaela will check everybody. Don’t worry, Michaela will check that we've received everybody’s feedback. Good! Well, that was evening one and it took a bit longer than I expected but I think it was because of the long introductions and everything at the beginning. Hopefully tomorrow evening we will finish earlier, but, participants and facilitators thank you very much for making it a very interesting evening this evening and I really look forward to seeing you tomorrow evening for our second evening of deliberations. So, thank you and I’ll see you tomorrow. Have a good evening’s sleep. Bye-bye, everybody!

**Day 2**

**First plenary**

Donrich: Greetings, everybody! Greetings! I hope that you’ve had such good and productive interactions than in my group. I was very privileged to have a very active breakaway group. So, let’s start with our feedback. The first group was Bongi’s group and if I may ask Participant 15 to report, please.

Participant 15: Yes, I disagree with the, I agree with the gene editing to be chosen to the child before birth because everything will be ok for the child when the child is born so the child will have a better life and most of us agreed to this and I will always agree because the times we live in has changed now and gene editing is really important for everybody. Thank you.

Donrich: Thank you, Participant 15. Then, in my group, if I may ask Participant 3 to report please.

Participant 3: So, in our group, yes, there was no consensus. Some people did agree that these diseases should be allowed, I mean, gene editing for these diseases should be allowed because, for example, if a person has contracted a disease, they would have to go on treatment for longer and may eventually come across pill fatigue. It was also listed that preventing children from becoming sickly is a good thing because their quality of life will be improved and also the financial help from the government towards the people will be reduced and so that was all for gene editing. And, yes, basically, so, if gene editing can reduce illnesses, everyone was agreeing to it. A very important point that was raised in agreement with gene editing was, let me see, oh, so that was actually against. So, against was a point that was raised was that for flu, for diseases that are not seen as serious, the flu, children may be, may not get natural immunity if they are just gene edited from the start, and we don’t know how that would eventually play out, let’s say, in the environment or in nature. Another point against, that was very unexpected, was that if diseases such as these that they can be actually prevented, because someone actually raised that wearing a mask, sometimes you cannot get the flu, but such diseases as well as HIV apparently, if you can control, your chances of getting this, then there's no point in investing resources in preventing the possibility of contracting these diseases in the future. So, but then, another point was raised against that that some people cannot control that risk, for example, rape victims or children born to mothers with HIV. And, yes, so basically it was against and for, natural immunity was raised, prioritisation was raised, pill fatigue. No consensus, maybe, I don’t know, if I left something out, please guys.

Donrich: I think you did a very good job at presenting both sides. If anybody in my group feels that they would like to present their views themselves, or if they feel that anything has been left out, they’re very welcome to raise their hands and I will also give them an opportunity. Good. So, let’s go over, thank you, Participant 3. Well done and let’s go over to Marietjie’s group. If I may ask Participant 19 to report, please.

Participant 19: Ok, so in our group we were pretty much in consensus from the very beginning. I initially felt that if we can prevent flus as well, we might as well do it. Let’s prevent everything that we can, but eventually, from being convinced by everybody else, they said that flus are very much manageable, the treatment is not long-term, the treatment is also very affordable. So, we eventually agreed on, I mean, flus and COVID and whatever else, should not really be a priority in terms of gene editing. And then, secondly, we were talking about more serious illnesses that carry a very high cost and a long term treatment, like HIV and TB. So, there was a point raised about how TB, sorry not TB, how HIV is actually preventable and it could actually be perceived as people that get it are irresponsible and the consensus we reached there was that science should not police the morality of people, and people will always make mistakes and if diseases can be prevented, then there shouldn’t be a sentence for something to, there shouldn’t be a sentence for being irresponsible or making a mistake. If technology and science can prevent it, then it should prevent it whether or not it comes as a result of being irresponsible. That was in addition to the fact that it’s not everybody that gets these diseases that was being irresponsible. Then, the same went for TB. The treatment is actually very long term. It’s not as long term as HIV, but it’s also quite expensive. There was also antibiotic resistance that was mentioned, because just because we have treatments for TB, doesn’t actually mean that everybody with TB gets cured, you know, we have MDR, XDR, all that, so it will be extremely useful if it could be prevented and then we wouldn’t have to deal with, whether it's MDR or XDR, or normal TB, no one would have to suffer from that. So, all in all, we all agreed that if something is serious enough, it has a long term treatment plan and is very costly to treat, then it should be prioritised higher on the list of gene editing.

Donrich: Mmm. What I am hearing is that there is now the issue of prioritising our allocation of resources. That seems to be a theme in your report. Am I right?

Participant 19: Yes, so there was an expression used that we shouldn’t use a hammer to kill a fly on the wall. So, we would have to weigh the costs, so it could be something that is allowed but there would have to be deliberations about costs attached to it versus the cost to treat it, we thought about productivity, how much the work force in a year would lose, and this was pre, this was assuming that gene editing for flus would make somebody immune to all strains and all future strains of all flus or colds. So we were looking at the cost of, you know, you lose four days here, four days there and collectively, if this is now something that is rolled out to the public and anyone with a planned pregnancy can do this, we were looking at that. And then, it seemed like overkill to prevent, you know, people losing four or two days pay and preventing spending like R50 a year on treatment for flu. The hammer and the fly scenario, yah.

Donrich: Thank you, Participant 19, thank you very much. And, whoever came, well, that’s an interesting metaphor. I would like to go back to the first group, the facilitator, Bongi, advised me that his group had a bit of a richer conversation and he would like a bit of a further report, so may I ask Participant 24 to also report from group 1, please.

Participant 24: Hi guys, hi everybody.

Donrich: Hello, Participant 24.

Participant 24: In our group, for gene editing for TB and HIV, we agreed because we thought, even though there is treatment, the quality of life is not the same as not having the virus at all. So, that’s why we agreed that it will be a good thing for it to be applied and for a person to have a choice, to be given a choice to use the technology itself, even before birth because if I am HIV positive, I would want my child to be negative. I wouldn’t want my child to have the virus itself, so it’s a good thing. And when it comes to, should parents be given the choice, or should government say that, no, this is what should be done – parents should be given the choice to choose for themselves, whether they want it for their child, or they don’t want it. That choice should be given hundred percent to the parents themselves. Also, when it comes to COVID-19, there was a split where some of us felt that we should use it, even though there is vaccines right now, we still can, it’s still contagious, even though we get vaccinated and you still can get it. It might not be as severe as it was before, but for the fact that you still can get it and you still, because we are not sure, you might still die from it. So therefore, we said ok fine, it should be used.

Donrich: Yah.

Participant 24: When it comes to flu, some of us said no because flu already, there's treatment for flu, treatment for flu, there's less, you don’t have to spend so much, and then if you take better care of yourself. Then when it comes to your common flu, it should not be, a line should be drawn as to how severe the flu is, so that’s what we agreed on.

Donrich: Thank you, Participant 24. I also saw that there was another hand raised, Participant 20, Participant 20, would you like to add anything from your group discussion? You were also in group one.

Participant 20: Yes, I am also going to add on Bongi’s group. Yes, we agreed on TB because we said TB is dangerous and then it should be subjected to parents. HIV, we agreed, I think all of us we agreed to gene editing on TB [HIV] because I say that the society we live in is very dangerous. Women are being raped each and every day, so should we prevent that, then we can go to, we can take that route. COVID-19, there was a split because some believed there is a vaccination, however, others argue that the vaccination is not a cure and that we have seen COVID-19 has made a lot of effect, like from tourism industries collapsed, the community collapsed, we also experience a high death rate. So, on flu, on flu there was also a split because some believe that, some disagree because it’s going to be more like, you, we’re changing the entire person’s structure while the flu is more manageable and then we have been living with flu for many years and then it, to others, the flu was serious but then to some, it was not. And then I also argued that I would disagree with that gene editing to like, in flu because while I was reading I also picked up that gene editing can also cause unintended changes such as a loss of an entire chromosome so in that case I would disagree with gene editing in flu.

Donrich: Mm. Thank you very much, Participant 20. Just a general point there, I think our assumption, I'm very glad that you’re doing additional reading, and currently that is definitely the case. I think that’s why gene editing is not currently done in humans because there’s a lot of safety concerns. But for purposes of our discussion, our assumption will be that it will be sometime in future when it will be safe and effective. Ok, let’s go onto the, to Magda’s group and if I may ask Participant 22 to report please.

Participant 22: Our group, we didn’t reach a unanimous decision. Some of us were more in agreement with some of the points than others. Mainly with TB and HIV, most people, no sorry. I’d say just over half of us were in agreement, but mostly subject to conditions. And then for the less serious ones, we were mostly not in agreement. Actually, by large, we were not, we disagreed that it should be legal, we said that it shouldn’t be.

Donrich: Can you quickly tell us, with the first ones, what were the reasons, and then also with the last ones.

Participant 22: Yah. For the first ones, some of the reasons I remember were that, currently, it’s, treatment for TB and HIV is already being paid for by the state and to, if that’s allowed to be changed by gene editing, then that load doesn’t have to be carried by the state and that can be covered by the state instead. As for those, HIV being mostly preventable, it, as someone pointed out, it’s been many decades, it’s been a few, several decades and cases are not going down and it’s not being prevented, so to prevent it permanently with gene editing could be a very good solution to stop new cases being spread. Against that were, I think the arguments were largely that it is preventable and for instance with TB, with a course of antibiotics that can be solved easily in six months and against that was that antibiotic resistant strains do exist and those, obviously won't be a six month quick and easy thing. So, to prevent someone going through that may well be worth it. Then for the cases for and against COVID and flu and the cold, the major one against was that it was to, as someone pointed out previously, actually, one of the earlier groups, I think it was group one, that it may affect your immunity in ways that we don’t know, so when you don’t, if you don’t catch the flu or a common cold when you’re a child, you may not develop immunity, you won't develop natural immunity to those and that may affect your immunity to other diseases, it may effect things that we don’t know or understand quite yet. Those were the major points. Another one I remember from COVID and the flu and colds, was that it can still affect your life, they can still be deadly, so similar to a vaccination, if you can prevent it entirely, well, prevention is better than cure, so yah.

Donrich: Good. Thanks very much Participant 22. That was very clear and succinct. Next to Meshandren’s group, if I may ask Participant 12 to report please.

Participant 12: Hi. Ok we have reached somewhat of a consensus. We did agree for the first, second and third one that they were life threatening conditions and that they should be prevented. For the fourth one, we did have a disagreement, me, I had a disagreement. I didn’t view the flu as a life threatening illness but the group members changed my mind. They mentioned swine flu and that could be life threatening and I decided to change my notion from disagree to agree subject to certain conditions. Yes, so that’s it.

Donrich: Thank you, thank you very much, Participant 12. Then over to the last group, to Ntokozo’s group, may I ask Participant 9 to report, please.

Participant 9: So, I think to just summarise everything, majority of the people, and they can chip in if I'm getting it wrong, majority was agree subject to conditions. So, some of the points that were raised were, is it particularly necessary? I mean, are we playing with evolutionary pressure itself? Because I mean, is it necessary for us to edit the gene line for things like TB, because I mean, there is already a vaccine, so, and that’s already working efficiently. I know that the conversation about cost will come up but essentially what we are getting at is are we taking away from the body’s own development in the sense that we are taking away its ability to develop its own defences against future diseases that might be similar to something like tuberculosis? And then with HIV, initially I had, I was actually of the view that we shouldn’t because it’s very much preventable, but Participant 17 did bring up a point and she did say that, something which I had actually missed out on. She did mention that there are still a lot of cases that are unintended, they happened by accident so for instance, someone is involved in a car accident and blood from an individual who is HIV positive squirts into someone who is HIV negative, or even healthcare workers, someone is injecting someone and they accidently cut themselves, cases like that. So I think, so we came to the conclusion that for that, it should be allowed. With flu we went back to the same argument of is it really necessary, are we taking away from the body’s own ability to develop its own immune defences?

Donrich: Good, well thank you very much, Participant 9. Participants, clearly there are some common denominators in the feedback. I trust that you’ve all observed these. If I can quickly summarise just some of those that seem to be quite pertinent to me is with the last one, the concern about, well, generally the concern about natural immunity. There's also a theme of prioritising, what is really important in terms of allocation of resources. But then also, the realisation that many of these illnesses are already a massive strain on government’s resources and that that must also be included in the calculations. So these are just some of the main observations from my side. So we will now go into our second breakaway groups. Again, I must just remind the facilitators to please record. Michaela, if you can put us in our breakaway groups, please.

**Day 2**

**Second plenary session**

Donrich: Welcome everybody, I hope you’ve got a, that you had a productive second breakout group. I see a few smiles, and Participant 22 is nodding, that’s good! So, let’s start our report back. So, let’s do it in reverse order this time. Usually I begin with Bongi’s group, this time I’ll begin with Ntokozo’s group. If I may ask Participant 21 to report back, please.

Participant 21: Hi, ok. So, one of our main topics of discussion, we seem to be pretty much in agreement that if it is safe, we are in favour of it. However, the bigger thing we were looking at is accessibility issues and that it has the potential to broaden the divide between the haves and the haves-nots if we just allow it to be accessed by everyone, well, by only those who can afford it before it is available and accessible by everyone. While we were, our discussions were moved on away from the specific diseases and things to more of a broader use of the technology. We also touched on human identity and how this sort of lucky packet pick of, well, not lucky packet, just sort of, pick, the condition traits, how it might impact our humanness as it were. There was a feeling that someone should look at it in terms of whether this is going to impact downstream generations in the sense that it might, we might be looking at whether altering human ability to respond and react to these diseases and conditions, if it has unforeseen consequences down the line, even generations later since it is a heritable technique. But, yah, so the main consensus we had in our group was, we are actually in favour of it, however, access is a big issue and that it shouldn’t just be for a few elite people, it should be, if it’s going to be accessed, it should be accessible by anyone who wants it and that it should definitely be something that we weigh risks against because if it is not a proven safe technology, we are playing with fire in terms of it having downstream negative effects. We also considered how it would affect our own ability to obtain immunities to conditions like the colds and flus if we are not allowed in the beginning, if this is something we want or if we are now just, it’s just simply a way of accelerating what we already have in terms of obtaining natural resistances and immunities if we, the gene technology is accelerating what we’d normally get to or is it something completely novel that now replaces it instead.

Donrich: Thank you, Participant 21. Anybody else from Ntokozo’s group who would like to add anything to what Participant 21 said?

Participant 9: I think also, something that came up just before the breakout room closed was, I think the best way to put it, so, Participant 3 brought up a point, the best way to put it would be, what kind of regulation would be there? Because if history has shown us anything with human beings, they always tend to take it a step too far and people start to almost like, play around with this technology so in the end you could have people trying to basically, almost, design a baby that’s going to eventually grow into a human being that can’t relate to other people emotionally, that’s almost as close to a robot. So, basically the big question is how far we go with this technology, when is the stop?

Donrich: And do you have an answer to that?

Participant 9: Currently I don’t, I just thought it was something that I could put out to the group. I’m still also thinking about it.

Donrich: There are many philosophers and ethicists who struggling with exactly that question, and they don’t necessarily have an answer as yet. But regarding...

Marietjie: And I think it’s tomorrow night’s topic of discussion, so I am looking forward to that.

Donrich: Indeed, indeed. Participant 9, but while I have you, just to make sure for the four questions, there your group are in, let me just understand, just summarise for me again.

Participant 9: We are in favour, well, actually there is a bit of a, so, the one sticking point is accessibility. So, what we...

Donrich: Yah, Participant 21 specifically referred to that.

Participant 9: So we are in favour, but we all did come to the consensus of, with accessibility, if it’s not going to be accessible to everyone then we don’t think it’s something that just a select few, group of people can have just because they have the capacity.

Donrich: Mm, thank you. Thank you very much, Participant 9 and Participant 21 for your reports. Let’s proceed to Meshandren’s group. I must tell you, well, my group reached consensus very quickly, but perhaps I was just lucky, but Meshandren advised me that in his group there was quite an interesting debate and he suggests that I give a few people an opportunity to first-hand convey their opinions, so I will start with Participant 14. Participant 14, if you can be first, but given that I am going to give more than one person an opportunity to speak, if I may ask you to keep it as concise as possible, please. Participant 14.

Participant 14: Ok. My concern was around posterity, potential rights being infringed, and also the technology being used as a panacea to cure everything or maybe similar to building a Tower of Babel, you know, where at one point it will collapse and we’ll find ourselves relying on one system. But nonetheless, it is an exciting technology, brings excitement and fear at the same time. Those were my, the issues that I face. I imagine the future generation bringing a law suit in 20-hundred and something over the issue of their human dignity being violated by the previous generation, you know.

Donrich: For being gene edited or for not being gene edited?

Participant 14: For having their human, I'm imagining this, probably a religious NGO at one point or whichever NGO, because naturally we've inherited, we've inherited genes on a natural scale. Now, by doing this it means that they would be inheriting genes that are edited, you know, so I'm imagining how it would feel for them, will it be, will it sit well with them? Will they be ok with it? I'm imagining at that point there will be a group wearing t-shirts that say ‘100% human’, and the other ones, you know, like, two opposing systems, more like Batman and Joker, something along that side, something along those lines, you know. Those are really my only concerns.

Donrich: Thank you, thank you, Participant 14. Those are definitely cause for further contemplation. May I then ask some of the other group members also. First Participant 22 and then Participant 6, please.

Participant 22: The rest of the, the group’s opinions generally were, agreeing and a couple said agree with conditions to the less serious ones. Very interestingly, some of us agreed completely with Participant 14 but we still differed in whether we’d want to allow it or not allow the gene editing. My view personally was to agree with all of them because they can all impact your quality of life and especially serious diseases, if you don’t, especially if you are at risk for them, if you don’t allow gene editing to prevent them, you may not be able to stick around or be able to pass on future generations, who then obviously can't object, so yah. It's a complicated issue.

Donrich: Mmm. I think we must realise that it is very complicated, especially given issues such as those raised by Participant 14, but eventually, of course, as a country, we must make a decision whether to allow it or not, of course. Participant 22, thank you very much. I will give Participant 6 an opportunity. Participant 6, do you agree? Is there something that you would like to add?

Participant 6: I do agree with Participant 22 and the one thing that Participant 14 did bring up is that he said that he felt it should be a personal decision for the people that are doing it but he still disagreed with all the points, so none of them should be allowed.

Donrich: It’s interesting how the same, let’s say premise in an argument, or the same set of facts, can be interpreted differently by different people. Thank you, let’s go onto Magda’s group. May I ask Participant 28 to report please.

Participant 28: Ok, in our group, we sort of agreed like the other groups as well in terms of the serious diseases and illnesses that gene editing is necessary there and it should be done. I think disagreements came on the COVID-19 and the flus which are not so life threatening. I think some of us felt that COVID is new, we don’t know much about it and let’s sort of nuke it if we can. But other people felt that our immune system has shown that it can handle it, there is a vaccine in place, it’s not so deadly, why are we even going there, let’s let our immune system deal with that and sort of learn to deal with other things that might come in the future. And then on cases of flu and common colds, it’s really, really unnecessary. Most of us agreed on that. And, we did have cases where some people were concerned about social issues like the other group was saying, if it’s available to some people and not to everyone, it might cause divisions and inequalities. I don’t know if gene editing is here to solve those problems, but there were concerns like that.

Donrich: Thank you, Participant 28. Thank you very much. We will now go to Marietjie’s group, and if I may ask Participant 7 to report, please.

Michaela: Participant 7 has lost connection, may I suggest that we ask Participant 26?

Participant 26: Ok, so, I think with our group, I may ask that my fellow group members also pitch in where I fall short in terms of presenting the discussions. I think there was consensus that if there's anything, it does not matter whatever disease it is, if there is a possibility of preventing that, then we should do it. But initially, I think that was the final decision of the group, or the final agreement, but initially there were discussions within that and they centred mostly around, firstly, in terms of immunity, you know, if we are, you know, using gene editing for things like the common cold, and we know that our immunity is perfectly able to handle that, to depriving ourselves of exposure to these non-deadly pathogens or viruses, whatever the case may be, we might just be damaging or disadvantaging ourselves in terms of our immunities. And, so, you know, we had quite an exchange around that, but ultimately, I think we came to a decision that says that this is ultimately what human beings do in terms of us manipulating nature and trying to find ways to, you know, improve ourselves. So, even this is also an extension of that, our evolution, so to speak. So, also, one other point that was raised was how, for example, how you know, if we, the assumption was that you are preventing a, you know, diseases like the common cold, it would be against all foreseeable strains, and we also know that there are other diseases and other pathogens that more or less share the same antigens or whatever the case may be, with the common cold or with these other viruses, so it might just be also conferring yourself an advantage in terms of preventing those as well. So, there was also a discussion about how gene editing is an advantage and should be done for, you know, should be encouraged because the environment currently, now, is exposing us to new and more deadly pathogens, you know, examples were cited like, you know the melting of the ice caps, which brought up new viruses which we have never seen before and we are potentially vulnerable to that, you know, the loss of nature and biodiversity, rainforests and all of that, us tampering with animals and all of that, is exposing us to pathogens, so, actually, us going more along the road of gene editing, that’s actually us protecting ourselves against these harmful elements of nature. I don’t know if I'm still audible?

Donrich: Yes, you are. The video froze, I don’t know why. Oh no, there it is fine again. But we can hear you properly.

Participant 26: Ok, I was also worried about that as well because I froze. I think the other thing that was discussed was the social issues and I think the consensus was more or less what the other group and what Participant 28 had said, that we don’t necessarily think that gene editing will be a panacea to solve all our problems, but also we recognise that science does not operate in isolation. It is informed by society and, you know, there is that vice-versa relationship where society informs science and science informs society. So, what we should be more careful about is to how we then not further divide society through science but rather make sure that the technology is equally accessible to all and we would make sure that we guard against the abuses of the technology. We did not explore a lot in terms of the human identity of how, you know, more and more gene editing might be impacting on what it means to be a human being. That was posed by the facilitator, but we did not, you know, go into deep discussions about that. What else was mentioned? But I think in summary, I think that in terms of all the scenarios that were posed, the group was highly for the use of heritable gene editing.

Donrich: Thank you, thank you, Participant 26, thank you very much. Very eloquent report back. So now over to my group, where Participant 16 will report.

Participant 16: Yes, thank you. So, I think we reached a lot of consensus actually in terms of most of these. For TB, we did say, based on the fact that TB can be such a serious, it is preventable, there is an antibacterial course that you can, an antibiotic course that you can take, but at the same time, it can lead to resistance and all of that, so in that sense, it leads to pressure on the health system and whatever. And I think we also agreed that if we can eradicate TB, then we can sort of remove that pressure in the health system and then, you know, to free up for more research into more diseases and all of that. So, if gene editing can be done in terms of TB, then we can, you know, do it. If it is agreed, then that will be the condition that we look at – the prevalence of it. You can't just make it universal, because in some places TB is not as common as it is in South Africa, for instance. HIV, there was a bit of a perspective in the beginning, like, it’s totally preventable, it’s a lifestyle disease, but at the end of the day, exploring more ways that this disease can be contracted, we decided that if we can protect the future generation against that, then that should be something that we should go for, and obviously also based on the prevalence of the disease in the nation. Like, South Africa, or sub-Saharan Africa would be a perfect country to do it because it has one of the one of the highest chance of that. COVID-19, I think COVID-19 through cold, we have seen that the immune system can fight against those, and that’s ok, but at the same time, they can be serious or they can be severe. We do know that more people die from the flu than they have from COVID-19, so that could be an argument for it, but I think majority of the group agreed that, maybe for the exception of COVID-19 because not only does it affect your health, it affects your livelihood. We've seen economies collapsing, on the bright side, you know, less carbon dioxide [laughs]. We've seen entire economies collapsing, there are still flights that are grounded at the moment because of that, they did not recover, so if we can eradicate not only COVID-19, but any possibility of future outbreaks, then that’s definitely something that we should explore. Especially living in such a globalised world, because my thinking was just do it in China, because they are the ones that have a new strain of flu every two minutes, just do it in China. But because we live in such a globalised world, I mean, two weeks later it will be in South Africa and then, well, you know, it’s basically like COVID-19 all over again. So for diseases like COVID-19, if we can prevent outbreaks, then that’s something we can explore, but for flu and colds, I think the consensus with the group was that you can prevent these, you can take your vitamin C, drink your water and all of that and you should be fine. And at the same time, we do not want to completely remove the whole immunity and I like the point that was mentioned about ice caps. [laughing] We don’t know what we’re going to get later and maybe having some form of natural immunity will help in the future generations as to fight against those. I'm not sure if I'm missing anything but I think I think I summarised it well enough.

Donrich: Thank you, Participant 16, thank you very much. Anybody want to add anything from our group? [silence] Good, then let’s move to Bongi’s group. If I may ask Participant 5 to report, please.

Participant 5: In our group we had a bit of a split. With the three questions, or the four questions, they all agreed that it should be used to advance the society, the medical people, the science, everything. I did not agree with that. So, basically, why I didn’t agree with it was because gene editing for me, the goal should be to give a person that do not have a choice and opportunity to start off on the same footing as a person that is born healthy. And his lifestyle and his decisions and using his opportunity and contracting something, that is the result of life. But the guy that doesn’t have the opportunity to choose a heritable disease or a serious disease or a less serious disease, those people, gene editing must be prioritised to help those people. Society and all the surrounding, the environment, everything, that shouldn’t be a factor in deciding what to use gene editing for, but my group definitely agreed that if they can use gene editing for all those questions and all those sickness, TB, AIDS, HIV and COVID and all those, yes, use it. But, I do not agree with that.

Donrich: Do you think, if I may ask you, do you think if you had more time, you would be able to convince the rest of your group of your position? Or don’t you think more time would have assisted?

Participant 5: Well, if I have more time and I could read some a bit of the research that’s already been done, definitely yes, but where do you draw the line in deciding when and for what are you going to use the gene editing? If you are afraid to live your life, maybe I contract something and I die of it, now I want to use gene editing, where are we going to draw the line of making people live a life? We will all be super and we can't die, we can't contract any disease, we can't, what life is that? Immune system is unique and immune system can protect you if you choose to live your life with the opportunities and you live it correctly.

Donrich: Mm, mm. Well I thought, given that your opinion seems to be a bit different, I encourage you to speak a bit more so to give it, well, to give you a bit more of an opportunity and well, thank you and I see that two people have their hands raised to, I assume to respond, so I welcome a bit more engagement regarding this. So, Participant 19 had her hand raised first and after that Participant 22 please.

Participant 19: Yes, while the group, I was in Participant 5’s group. While the group fully respected and understood the merit of Participant 5’s argument that people that don’t have a choice, who will definitely be born with those diseases, they should be prioritised and that gene editing should help them, we also realise that the incident of people with a genetic defect, I'm sorry to use that word, diseases, there's a very low incident of those people and the communicable diseases, the ones that are perceived as ‘we have a choice’ or ‘there's a probability that you’ll get it, and you might never get it’. Those have a higher economic impact and social impact. We understand her and fully respect her stance but we feel that very few people, while we agree that they should definitely be prioritised, we fear that limiting the use of such an important tool to only those like diabetes or Down's syndrome, you know, all of those, Alzheimer’s, so it would be extremely limiting something that could do what science has been all about, science and medicine has been all about all these years, namely which is eradicating diseases that impact the economy so, so much. If you think of TB, and how much is being spent by individuals and the government collectively to cure it. It is true that a great proportion of our population will never contract TB, but it is also true that the impact is there and the impact is extremely severe. So, we did respect her stance and we fully, fully agree that it has very good merits, but we firmly stand by serious diseases, or the gene editing being used for those serious diseases because we believe that medicine and science and technology have the responsibility to prevent bad impacts.

Donrich: Mm, mm. Participant 19, thank you very much to both you and Participant 5 for your feedback to the plenary. Let’s conclude with Participant 22, please.

Participant 22: Sure. Sorry, just, Participant 5, you said something that I would like to ask about. Specifically you said something about how if we never got diseases or illnesses, then life would be very different. But you seem to be phrasing it as a negative. I don’t see the down side. If you could never get diseases, I would quite welcome that, actually. Can you just explain your view on that, please.

Donrich: Thank you, Participant 22, and then I will give Participant 5 an opportunity to briefly respond. Participant 5, over to you.

Participant 5: What makes life is actually what you get in life. If you have colds and diseases like flu or TB or things, like, that could be prevented, yes that makes life’s journey more difficult. If you do not have it, what are you going, what is your experience then, through your life, in a way of your immune system? If you go and you change your immune system and you have no diseases, not at all, who’s to say that new ones won't appear that is more aggressive to immune system? What are you going to do then, when you’re thirty? You had this editing when you were five days old, now you’re thirty, now there's a new COVID that was never in your immune system. What are you going to do now? Now you have nothing. You have no, your immune system have no memory of any disease, or how to handle a disease. How are you going to live now? Who is going to help you now?

Donrich: I will allow one last response. Participant 26 raised his hand, so Participant 26, over to you and then we’re going to do the poll. Thank you very much everybody for participating so actively, it really makes for a very informative and interesting discussion, but Participant 26 will be our last speaker. Over to you, Participant 26.

Participant 26: Thanks, I’ll be brief. Maybe I’ll pose it as a question, I know there are very knowledgeable people here. So in terms of, for example, if I get a vaccine, like, if I get a vaccine, that’s basically me priming up my immune system such that when I do get the actual disease, I'm actually protected, you know, my body, you know, can fight or amass a response against that, right? So I believe also that the same, more or less the same, could be done with gene editing such that you can in a way, of course, that’s why I'm posing it as a question, some form of, you know, such that when you do edit those genes, but you also confer an ability to amass an immune response should that disease, because I think that’s what is, in terms of infectious diseases, that’s what actually one of the pathways that it could go down in terms of, you know, with actually affecting you not getting HIV. In saying that when HIV comes to you, you already have immunity against that. So, I don’t know if that makes sense.

Donrich: I understand what you’re, well, I think, if we can resolve this in the following way. I am trying to make it simple. In the questions that we’ve, well, in the way that we have formulated the policy questions, we have stated that, we assume that the technology will be safe and effective. So, I think we must just remain with those assumptions that we’re making in terms of safety to one’s health. I would like to conclude this now, but, Participant 9, can you add value, can you provide us with more insight? I see your hand is raised.

Participant 9: Yes, just in response to Participant 26’s question, or rather what you were saying, so, to basically provide immunity and then confer the ability on the immune system pathways that actually fight these diseases, it’s quite a, it’s quite a tricky subject because it’s, so, particularly with the HIV one, it’s something around what I worked with in my Honours project last year, and you can, so currently there are people who do not have, so they have a mutation that deletes a receptor called the CCR5 receptor, and that’s the receptor that your HIV virus actually binds to. So, people who do not have that CCR5 receptor, some of them have been shown to have the HIV virus in their bodies, but they show no signs of infection. So, you can delete it, but then, I don’t know if it’s possible for other things like flu. What exactly are we looking at? Because there are different strains of the virus.

Donrich: Well, that’s very insightful and thank you for sharing that. It’s good to know that there are people with thorough genetics knowledge here among us. But, I think I will, I think, as I said, we keep it simple. The assumption in the question is, it will safe, it will be effective, and we work with that. So, at this stage, we need to cut the conversation and go to the polls, so I am going to launch it now. Michaela, if I can, I see the poll is yesterday’s poll…

Michaela: At the top, where you see the title of the poll, there should be a dropdown arrow.

Donrich: Thank you, thank you, it’s always good to have a young mind here to assist me with technology. Here we go! [silence while participants vote] We’re waiting for one last one.

Michaela: Prof, we are at a total of 28 so that’s all the participants that are present at the moment, one of them lost connection.

Donrich: One hundred percent. Ok, everybody, let’s have a look. I'm not going to speak about it, I'm just going to scroll through it. I imagine you can see it on your screens. Michaela? Ok, so that’s number 2, very similar to number 1. There is number 3 and there is number 4. Thank you very much. So, to all our facilitators, all our participants, this has been massively interesting to me and thank you very much for everybody for engaging, for sharing your opinions and for doing so very respectfully. As researchers, we really, really, appreciate your time, your effort, your participation. So, this is the end of our second evening. We’ll see you tomorrow evening for our third and final evening at 18h30. Please, be in time, we start at 18h30 sharp. Thank you everybody. Enjoy the evening. Bye-bye!

**Day 3**

**First plenary**

Donrich: Greetings, everybody! And welcome to our first plenary report back session for evening number three. We could hardly, we could in fact not finish our conversation in my breakaway group. I hope that other breakaway groups were more successful than me. So, let’s hear the reports back. Let’s just mute ourselves please. Michaela if you could just mute all, if you don’t mind. Ok, great. Good, so first if I could ask Bongi’s group to report and there, Participant 26 is, Participant 26 has got the microphone. Over to you, Participant 26.

Participant 26: Ok, so on the first question, of intelligence and athletic ability, we mostly agree that it should be allowed, but there was also some, you know, some division there on when, for example, the choice would be then, what does athletic ability, for example, confer as an advantage to the human race per se, as compared to intelligence? So, we thought that those were two different categories. So, whatever traits that we see that are beneficial to humanity and to progress, like intelligence, which is applicable to a wide domain of things, then if we have an opportunity of improving that, then we should do it. So, I think that was the first point where we agreed on, we mostly agreed on. Then on the second one, so other reasons why people agreed was that for example, it’s inevitable. If people, if there is gene editing, it might as well, and the law doesn’t allow it, then we might find that people, you know, use illegal channels, for example, to try and do that. And when it’s being done in that manner, then it opens up, you know, a whole can of worms. Then, on athletic ability, for example, we contrasted the two, intelligence and athletic ability.

Donrich: Mm, that’s interesting.

Participant 26: Yes, as we understand that they fall in different categories. So, for example, in athletic ability, there is a danger there that maybe some, we have the creation of super soldiers for example, or people who are so augmented or who are, who have unfair advantages over others, for example, in Olympic games or in competitive sport and things like that. So, and also, you know, so, that would be, you know, things that we would not want to have, so, you know, that’s why you categorise the two. But also, another point that came up was the fact that there is also a concurrent discussion of artificial intelligence and if we want, you know, and that’s where we are moving as the world. So, we would need, I would say, that human intelligence to be at more or less the same level, or you know, so that we can be able to better understand and fully use and integrate with AI. So, on the one of personality traits, there was disagreement with that it should not be used because it undermines human agency. There might be a creation of, you know, find a sense that, it is used for human control, you more or less create zombies and that can also have the potential of being weaponised, such that maybe you create these aggressive people that you can maybe let loose, or whatever the case may be, on a particular… we know the possible scenarios with regards to that. And also that people would lose their individuality and it would more or less take away human choices, because some of these things, as much as personality is inherited to a certain degree, but it also goes according to the environment, environmental conditioning, how people are raised up and also, you know, choices. So, by doing this, we would essentially be undermining all of those things. On the one of sexual orientation, there was disagreement, we all, there was consensus on disagreement. It was that to a certain degree, sexual orientation is a choice, and you know, if, again, the same points of controlling human behaviour and making people what you want them to be or controlling, making people zombies, was also brought up here as some of the reasons why we shouldn’t, you know, allow the sexual orientation to be something that can be modified by the genes. Then in terms of skin tone, the consensus was, again, was that we disagree, because it’s cosmetic. Neither provides any benefit to humanity, nor does it alleviate any human suffering or, and what becomes trend in one era might be forgotten trends in the next. Blue eye colour might be a thing now, the next it might be brown colour and all of that, so it’s cosmetic, it’s of no use. And also, for things like skin tone and all of that, it might just be opening a door to things like eugenics and where, you know, it might just expose people’s, what do you call, underlying prejudices and all of that. And also, it might also have a negative influence on the diversity that is already apparent in our world. So, I think that’s a summary. If I have left out anything, then my group members can pitch in.

Donrich: Well, I think that was a very eloquent and interesting report from you, thank you very much, Participant 26. Let’s move to my group, if I may ask Participant 23 to report please.

Participant 23: In terms of athletics and number 3 and number 4, we all disagreed. We said that let us not intervene with the nature and said it’s extreme, it’s a [inaudible] of the person so people shouldn’t, people should accept the way they are. And then, as much as we want what’s best for the child, it is wrong for a parent to also, like, you know, want what’s best for their child and then edit their genes. And then in terms of intelligence and aggressive, to aggressive we said only if it has been discovered in the brain if the child is having a problem, then it can be allowed for, to correct this, the same gene, but only if it is handled correctly. Also with the intelligence, it’s subject to conditions, you know, only if there is a problem that has been discovered in the brain then yes, it can be allowed, but subject to conditions. I think that’s all, but if I left anything, then other members can step in.

Donrich: Thank you, Participant 23, much appreciated. Let’s go over to Marietjie’s group. If I may ask Participant 27 to report, please.

Participant 27: Hi! Our group came to a split. Most of us disagreed, some of us disagreed [agreed] with subject to certain conditions. And, one of our members agreed that it should be allowed, but parents should have a choice to choose what they want for their child. In terms of intelligence and athleticism, I think that most of us disagreed. For personality traits like aggression and being cooperative, most of us also disagreed. In terms of sexual orientation, most of us disagreed and for determining aesthetic characteristics, such as skin tone and eye colour, I think we all disagreed with that.

Donrich: So tell me, what were the reasons? What were the main reasons for disagreeing with those?

Participant 27: For disagreeing with intelligence and athleticism, I think that, this is my personal point of view, I'm not sure if I can remember my group members’ opinion. But I would say that if you can choose intelligence and athleticism, it would be more of customising your child, or creating your own version of what you want the child to be, instead of allowing the child and accepting the child for who they are. In terms of personality traits like aggression and cooperation, I don’t think that people can be viewed in that dichotomy of either being a hundred percent aggressive or a hundred percent cooperative. We all fall in a spectrum and I don’t think that you can actually pinpoint and say this person is a hundred percent aggressive and must be edited so that they can be cooperative. In terms of sexual orientation, that’s something that I would strongly disagree with and I would say that people, whether it’s parents or children, should be allowed to have the right to be who they are and it’s part of being human, just as much as each person can choose what they want to eat, or what clothes to wear, they should be able to choose who they love, how they want to live their life and to be happy with the life they live. I wouldn’t want to imagine children being born living a life where they are forced to be someone who they aren’t, where they are forced to be unhappy, live an unfulfilling life, just because of the opinions and thoughts of their parents, their society, their religion. I would want it to be a fair and equal world for each person.

Donrich: Thank you, thank you very much. Thank you, Participant 27. Let’s move to group number 4, Magda’s group. If I may ask Participant 25 to report for us, please.

Participant 25: So, in my group I think we unanimously decided that we would not agree with anything. So, for instance, with your talents, we feel as though intelligence does exist in a broad spectrum and if parents are given the opportunity to decide how intelligent their baby should be, they would obviously opt for the super intelligent end of the spectrum and that would obviously mean that we do away with, you know, things like conflict resolution. What conflict will there be in society? You know, I think intelligence kind of compliments, you know, intelligence is complimentary, right? So, for there to be decision-making and conflict resolution, we should complement each other, right? Then with personality traits, we definitely disagreed but we might be open to agreeing if, or rather, in instances where a child, so, after their genetic screening, we definitely are sure that they will be serial killers, so in such instances, we could be open to the idea. Then with sexual orientation, definite no as well. And then with your aesthetic traits, again, I think it will just, you know, perpetuate certain stereotypes in society, certain constructs, such as beauty constructs in society and issues such as racism and all of those other things. So, the other thing that did come up is the issue of it being reversible, so if we do opt to, you know, confer a blue eye colour on a certain child, would that be reversible? If maybe in the future the child doesn’t want that eye colour, is that reversible? If not, yah, it’s really something else. And then, another thing that did come up with your talents, specifically with you intelligence is that, you know, in society we've got different socioeconomic backgrounds, so some people are from well-off families and others are not from well-off families and that obviously means that if we’ve got the same type of intelligence, or same kind of intelligence, and then how would the person who is not from a well-off family make it in life, you know, because the other one has resources and on top of it, they’ve got intelligence, so they are pretty set off for life. So, yah, I think that’s just what came up.

Donrich: Thank you, Participant 25! I see that Participant 19 has her hand raised. Participant 19, over to you.

Participant 19: I was in that same group. We also discussed how doing that could be undermining free will because, for instance, with intelligence, it’s not just your genetic disposition, there is also hard work that is involved in that. With athleticism, the same thing. So, you might already have those good traits, maybe you have longer legs, whatever, but you still have to work really hard, so it would be a really unfair advantage, because even in situations where somebody is from a very well-off family and they have all the resources to, you know, to make it in life, they still have to make the decision, they still have to make that choice to work hard, no matter how rich your family is, you still have to work hard, so that undermines hard work, it undermines people’s choice and also we also thought beauty standards change over time. In ten years we won't have the same beauty standards. At some point wider hips were a thing, at some point narrower hips were a thing, so if you’ve chosen what you think is good for your baby now, what happens two years later when it is no longer the trend?

Donrich: Yah, yah. Thank you, Participant 19. Let’s proceed to Meshandren’s group. There I would like to ask Participant 20 to report please.

Participant 20: Ok. On intelligence we have three no’s and one yes.

Donrich: I just need to see your video, Participant 20. Yes, please. There we go!

Participant 20: Sorry. Ok number 1 we have three no’s and one yes.

Donrich: Mm, and what are the reasons?

Participant 20: As well as number 2, on intelligence I have argued that I would agree with gene editing because I believe we need intelligent generation, people who are going to lead us with great intelligence. On aggressive, I also agreed because we have a high crime rate and then number 3 and number 4, it was a no. Number 3, it’s more of changing the whole being, sorry number 4, it’s more of changing the whole being of the child, and then someone else also argued that diversity is also important and then we should also accept diversity. I can't remember well others, but I'm just going to please ask Participant 3 to add on.

Donrich: That’s fine, just tell us what you remember.

Participant 20: They had a valid point, ok. Number 4, it’s a no because of diversity. And then on sexual orientation there was also another, a lot of debate around that because of non-binary as well. Yah, I think it’s all that I can remember.

Donrich: Thank you, thank you, Participant 20. It’s really added to our knowledge for our debate. If anybody wants to add at any stage, you’re welcome to raise your hand, but I will then in the meantime proceed to Ntokozo’s group and there, if I may ask, or, first, before I go there, I see Participant 22’s hand, so Participant 22, it’s over to you.

Participant 22: Yah, thank you. I just want to expand on some of the points that were raised in our group. For talents, first of all, I guess, how many people, for talents, we had three no’s and one yes. Personality we also had three no’s and one yes. Sexual orientation, we had three no’s and one yes with condition that it has to influence people to be more, closer to nonbinary, so...

Donrich: Oh, that’s interesting! So, yes you can, but then it must be closer to well, nonbinary or bisexual? If you can just explain for everybody’s benefit, also for my benefit that I properly understand.

Participant 22: Sure. I asked the same. It was, I think, if you can change sexual orientation, then you can probably change what you identify as, as well, so then to change closer to nonbinary so that it’s more of a choice what you identify as. If I'm getting this wrong, anyone from my group, please correct me. But yah, I remember it being as, so it’s more of a choice what you identify as and yah.

Donrich: Ok so in other words, if it’s then allowed, if we assume that say, sexual orientation is on a spectrum, then it must be changed to the middle and not to one of the extremes of either completely homosexual or completely heterosexual, if I understand it correctly?

Participant 22: Yah, that’s what I interpreted it as.

Donrich: And, well, I think we, that’s definitely an interesting suggestion – Participant 27 raised his hand, but Participant 27, I think let’s just give Participant 22 an opportunity to speak about the other questions as well, and then I will give Participant 27 an opportunity to share his opinion regarding that.

Participant 22: I will quickly summarise the other arguments. Like the person in our group said, we need diversity, and that’s very important, so we all voted no. Then the other, other ones, for intelligence one of the arguments was that it, you may influence something that you’re not intending to. so, there's already a link between intelligence and depression and to try and increase intelligence dramatically may also increase depression dramatically and other things along those lines, for instance, you may decrease empathy a lot, if you increase intelligence. Personality, decreasing aggression can also have side effects, for example, you could decrease competitiveness, or it’s, you need aggressiveness, controlled, of course, but without being competitive, you’re not going to get very far in sport. Yah, that’s just the ones I think were missed.

Donrich: Great, thank you very much, Participant 22. Participant 27, over to you.

Participant 27: Hello everyone! I just had one question, in terms of question number 3, to influence the sexual orientation of a child. I wanted to ask, if sexual orientation is actually a choice or whether it is naturally something that someone is born with? And if it something that we are naturally born with, wouldn’t it be unethical to change a choosing?

Donrich: Well, I don’t want to have a debate about whether it is, whether people are born with a sexual orientation or whether there's environmental input to it, that is why when we formulated the question, we chose the word ‘influence’ and not, we didn’t say it will be determined, we just say it will be influenced, so it is your own, based on your own knowledge and or value system that you can decide whether you think it is purely genetic, whether it’s environmental or whether it is a combination thereof. So it was a specific choice on our side to just say ‘influence’. Siddharthiya, we will appreciate your input.

Siddharthiya: Sorry Prof, I just wanted to say that, we’re assuming in these policy questions that there is a genetic component, but obviously we know that there's a nature versus nurture argument, so, yah, I think all the policy questions, there's always a nature versus nurture argument. We’re just assuming that in this case, if science were to say well, this is what we found, in that case, how would you feel about it? I think, if I'm getting that right, Prof?

Donrich: Yes, yes.

Siddharthiya: And just on the, I think on the note of, the sexual orientation, how a person identifies and who they’re attracted to may be two different things, so that’s why nonbinary and bisexual have a difference. Nonbinary is how you identify, whereas bisexual is to whom you’re attracted, so just to keep that in mind as well.

Donrich: Thank you, Siddharthiya. It’s good that [young people] can clarify these things for us. Participant 20, over to you.

Participant 20: Ok, I just need to add one thing on why we said no on number 4. Ok, having said that we have changed the baby’s skin tones, we have changed the colour of the eye, maybe you have changed the child to a Kim Kardashian style, someone said in the group. And then what is going to happen in the years to come if that change that you have done is no longer the in thing, like it’s no longer a trend?

Donrich: Yes, that’s if parents got themselves led by a specific trend at that stage. Thank you, Participant 20. Let’s go over to Ntokozo’s group and ask Participant 17 to report, please.

Michaela: It looks like Participant 17 has lost signal, as far as I can see.

Donrich: In that case I will ask Participant 1, if you please.

Participant 1: Yes, in our group we, majority agreed no and we had one person that said yes in all cases, he believes that it will happen either way and he had a nice thinking of, this is how the government can make back the money that they have to spend on gene editing for the more serious diseases and he also felt that the parent has the choice. He also felt that if it’s like your second or your third child, if your first or your second child had some intelligence impediments, that you can have a baby that could possibly look after or care for that all the siblings, so in that terms it would be a designer baby. The reasons for disagreement was in the intelligence was our population is diverse and yah, you might just widen the social gap in some instances. With the sexual orientation thing, it was, ok, I felt that why would a parent need to have a choice? What benefit is there for it? And also, you are now stepping on people’s rights because people have fought for these rights in the country, so how are you going to, you know, say that it’s legal and yet you had people fought the battle hard for their rights? With the skin tone and the eye colour thing, it was also, there's already biases in society that lighter skin tone, doesn’t matter which race, always will get a job or will go ahead in life, so you will just push that forward. I personally felt that that is against what the Constitution is going to and then you are also going into the phase of building this perfect looking, yah, so I felt that it was a bit of a dictatorship. Yah, so we said that the intelligence and the other point was with the point number 2, that you can influence the genetics, but there's still nurture, and nurture has a big impact on how your child grows up. So you might eliminate the genes, but whatever happens to the child when that child is growing up will also impact, so it might be a futile exercise. But I like Participant 13’s thinking of, it could be a way to make money back. [laughing]

Donrich: Good! And thank you for your balanced report there. I am quickly going to summarise the points, I've been making a few notes here and then if anybody thinks that I've left out something that’s important to them, then they can raise their hand. So, I just scribbled down a few key concepts. So, people highlighted the value of diversity and they’re afraid that if some of these are allowed, it can compromise diversity in South Africa, in our society. The value of free will has also been highlighted, and the fact that nurture is important and together with that hard work to accomplish things, is also a value that we should strive to protect. Also that some of these possible changes may have side effects that are not necessarily intended, such as higher intelligence might be associated with more depression and aggressiveness, or at least a measure of aggressiveness might be good for competitiveness and the positive things that we then associate with it. On the other side, there's also other values such as parental choice that was highlighted, that parents should have the choice. Also that we can improve humanity, that that is a value, improving human be allowing some, at least some of these choices. And also that society can benefit and that in fact, society needs more people who are smart and society, well, there must also be less crime in society, that might also be a reason for allowing this. And then, lastly, the argument that this kind of choice will happen in any event, does it really make sense then to try to ban it? So these are my notes, if you think I left out anything, now is your opportunity to raise your hand. Not as an opportunity to debate it now, but just if you think I left out any of the main reasons. [silence] Good! Well, this is massively interesting so you’ve now heard the spectrum of arguments. I would like you to now go back to your new breakout groups and this will be your last breakaway group for our study and then really debate this in detail. Try to see where you can find consensus. Is there a possibility of consensus between these wide ranging views? Really explore what these values really mean if you speak about these kind of things. Engage with each other’s values. So, reminder, facilitators, please record. Michaela, if you can throw us in our groups, thank you very much! Remember to record.

**Day 3**

**Second plenary**

Donrich: Greetings, everybody, it’s our last plenary session! This was massively interesting, I must say. So, this time we will begin with Ntokozo’s group, and if I may ask Participant 10 to report for us, please.

Participant 10: Sure. So, in our group we basically we went through and had a chat about whether we felt that our opinions had changed based on what we’d heard. For most people, that was, the answer was no, we still pretty much stick to what we had originally, what we had originally said. We did have one person say, well, ok, maybe with regard to the intelligence question, you know, if we could see ahead that genetic, well, gene editing could be used to see if a person was going to be stuck with a learning disability or something like that, that we could correct it and that wasn’t something they’d thought about previously. But for the most part, our group remained pretty set on what we discussed and we’re pretty unanimous, pretty much a consensus on what should and should not be allowed.

Donrich: Thank you, Participant 10. Thank you very much. So that was the report from Ntokozo’s group. Next is Meshandren’s group. Meshandren advised me that there was a very lively debate regarding issues of identity, spirits and so on. I really look forward to hearing that. May I ask Participant 18 to report please.

Participant 18: Hi everyone. So, with our group basically people are still firm with their beliefs. I think it was Participant 21 who kind of changed his for the first two, with certain conditions. And then the subject of identity, so, yah.

Donrich: If I can just pause you there, on question 1, what was the thinking on question 1?

Participant 18: On question 1, from what I recall, everyone was agreeable to intelligence except for Participant 21, because he just felt it was eugenics all over again. Hello?

Donrich: Yes, yes, yes we are hearing you. And those who were in favour of allowing parents to decide that, what was the reason there for allowing it?

Participant 18: For allowing, I don’t really remember because we didn’t really focus on that, we really went in on the identity and then it still kind of…

Donrich: Ok, well then tell us about the debate regarding identity.

Participant 18: On the topic of identity, we were talking about free will and how our genes are connected to our spirits and, you know, there was an example made that, I got it from my mama, so would the child feel the same way, like, would she feel like she did get it from her mama, would that baby have that same connection with the mom or her family? It felt like we were denying the child of their history. I don’t know what’s going on here, my video is gone but ok. Yah, I think, if I could please ask maybe my other group members to elaborate further.

Donrich: It sounds like a very interesting debate, so, thank you, Participant 18. Would anyone else from your group like to expand on this, on this debate? Perhaps Participant 3?

Participant 3: I was listening more and, I don’t think I recorded well, I was just trying to be in the moment.

Donrich: That’s hundred percent. Participant 14, would you like to give us a concise summary of that debate?

Participant 14: Ok, I can try. Generally, for all the questions, it was no. Participant 21 mentioned around the issue of intelligence, he was more open to it, subject to certain conditions, though we did not dwell into the matter of what conditions could that mean. But Participant 3 raised a question around spirituality, you know, and I came in in a sense of mentioning there was an album that was released a couple of years back by a jazz artist who passed on in South Africa, I think it was Moses Molelekwa called Genes and Spirit and the question that always pondered in my head was why did this particular artist decide to call his album Genes and Spirit, and I'm still trying to find out why is it so. But, to a certain level of my understanding, I think that goes to one’s own identity, you know, genetically we inherit [inaudible] from both parents and spiritually, here in this particular, not necessarily here in the country, and I'm considering Australia, North America and so on, they also believe that there is a spiritual fibre that we connect through with our ancestors, you know, beyond genes. The issue of, you’ll sit around with family and a child’s behaviour would be likened to his or her ancestors. Her behaviour is similar to her grandfather, you know. So taking that away, it’s tantamount to wiping off her identity and taking off her identity, you know.

Donrich: I just want to understand you properly. So, what you are saying is that if we are allowing parents to genetically edit personality traits, behavioural traits, that will then impact negatively on that spiritual connection of the next generation with previous generations. Do I understand you correctly?

Participant 14: To a certain degree. Let me be clear. There was a documentary that I actually watched where this evolution scientist mentioned that he came across the issue of spirituality and he was working on genes and imagine that these are two separate elements. But, geographically and humanly, we identify differently. Someone would connect with their ancestors through the spiritual realm and naturally their ancestors, she or he inherited the genes from their ancestors. So, if, say for instance, a personality trait has changed, you know, a personality trait has changed on this particular child and they are sitting around fire or sitting around in the house and they do not necessarily recognise this particular identity, that means something else for this particular child and I'm saying this in the context of, say for instance, when a child is born, a lot of the time, before the child could be acknowledged from the father’s side or the mother’s side, there were certain secrets that the elders knew. They will look at certain elements in terms of how the hands look like, how what-not looks like and they will say no, this one is ours, we acknowledge him. Now imagine all of those elements have changed. And bearing in mind that this technology means so much. It changes everything. It changes how we see things, it changes how we perceive the future, it changes the laws. It changes almost everything. We’ve literally opened up the book of life, you know. So, the consideration should always be on the child’s interest, at any matter, even in South Africa…

Donrich: I need to ask you to make your final point now, so how does this then affect your decision regarding whether question 2 should be allowed?

Participant 14: It goes back to the identity, you know. Identity, it goes to so many things – names, how you’ll give a child a name. Sometimes you’ll give a child a name that was belong to his or her ancestor and somehow there would be certain elements that that particular child possesses that that previous ancestor possessed. It’s a lineage. It’s more of, it’s a history of communication. Its modes of communication, how the past communicated with the present, how the present communicated with the future and so on and so forth, you know. So, wiping off the default or the challenges on posterity, it’s tantamount to burning a library, but that child can have an option to alter his or her genes to counteract against certain elements without altering the modes of communications that have always been there. Hence I am saying, this effects the identity of humanity, you know, it’s more like wiping off everything that’s been written, it’s been, in short, it’s more like being ahistorical.

Donrich: I understand what you say but it’s not clear to me how you connect it to whether the state should allow parents this choice. Should the state then allow parents this choice regarding behavioural traits, or should the state prohibit parents from having that choice? In other words, I need to understand your observations, is that something that is the context around this, or do you think that has a specific impact on policy, or should have an impact on policy?

Participant 14: Certainly it should have an impact on policy and I don’t want to really become dictatorial and I am for people to have their own choice, but I'm just, this is more of a persuasive argument to say, I am assuming a position of being an advocate for the future. There's a legal invention that came in the 70s or 80s or 60s which was called nasciturus fiction, whereby the child’s interests were protected before they were even born. So, for me I don’t want to find ourselves being caste by posterity and saying, you know, father, mother you’ve took so much away from me, you’ve took my identity from me, you know.

Donrich: Participant 14 thank you very much, it’s been insightful but I think time is against us so we will have to proceed at this point. Participant 21 has his hand raised, so over to Participant 21.

Participant 21: Thanks, Donrich, I just want to clarify my own points here that were raised in our group. Basically, I was in favour of only using the technology or intelligence or behavioural alterations in cases of medical necessity, such as when, if a family history of aggression or subnormal intelligence, perhaps [inaudible] have a look at that, but when all the cases I was pretty much opposed to it, so I was more of a no, with exceptions position. Regarding the whole identity discussion, Participant 18 brought up that basically, which I do agree with, a child’s identity is something which they acquire as they grow up, and that even with the initial starting blocks, they will still then grow into their own selves. However, Participant 3’s point as well of connection with the ancestry and culture can be effected, especially if they feel or are different. She brought up the example of biracial children not being able to specifically find an in-group to identify with and how that can be seen as an issue, but, so yah, my position has always been that this is a bit of a eugenics type thing, so I am opposed to it in most cases with only the odd potential medical intervention.

Donrich: I understand. Thank you very much for the clarity, Participant 21, and for also sharing your reasons for that. We’ll proceed to Magda’s group, and there if I may ask Participant 22 to report, please.

Participant 22: Sure. I may need to just need to ask my group members, if I'm forgetting something or getting something wrong, please just correct me. So, most of our group, as I remember, three out of five disagreed for, actually, all four of the points. We had one person agreeing completely for all four and I think one person agreeing with conditions for the four. So, the main arguments as I remember were, for talents, that, well, for the people agreed, who said they support it, it was mainly that it’s going to happen whether we want it to or not, so whether we allow it or not, it will happen in other countries and we don’t want South Africa to get behind. Especially in first world countries, and we will end up behind them. Then, the other one was that we, sorry, I'm trying to remember it now. That we can, we have the power, if we have the power to change these sort of things, then why not use? Why not use whatever advantage we can get from it? We can better humanity by doing that. And then, against it were that we may effect far more than we think. So, to effect intelligence, like I said with the previous group, can affect your personality and can, for example, give you depression. To affect personality and aggressiveness can affect competitiveness and removing competitiveness is not just for sport, it can also have a very big effect on academics, so if you increase intelligence but decrease aggressiveness, no matter how intelligent you are, if you have no drive to get something done, you won't use it. Then sexual orientation, most of us said no that’s something that should not be changed by your genetics, as in, like, we should not change your genetics to change that. Yah, I know I am missing some points along the way but… Then aesthetics, for skin or eyes, as humanity, diversity is what gives us such strength and, yah, to, also, to change something and then later, either the child doesn’t like it or other people don’t like, would both have a very negative affect on them. So, if you give them something, and of course this applies to sexual orientation as well, if you give them something that they end up hating, they’re not going to have a nice time with that. If, for example, lets say you make your child gay. In a lot of religions, in a lot of cultures and some countries, that’s unacceptable. Some countries you can be killed for that, and completely legally even, so to be killed by something that your parents chose is not something you want to aim for. And those are the main ones that I remember. If anyone from my group can remember the other ones, please feel free to add on.

Donrich: Thank you, Participant 22. I see Participant 25 has his hand raised. Participant 25, would you like to supplement, to add to what Participant 22 reported on?

Participant 25: Ok, yes, so I think he covered pretty much everything, but I think with aesthetics, someone had said that if it is done earlier, you know, when the baby is still an embryo, then it would have lower costs on the mother or the parents, ok, so it would be rather nice, you know, just for the purposes of finances and all of that. Yah, I think that’s just the only thing that was left behind. Then, yah, yah, just that.

Donrich: Thank you, Participant 25. Thank you very much. Let’s process to Marietjie’s group. If I may ask Participant 12 to report, please.

Participant 12: Hi everybody! Yes, we came to like a disagreement on most of the subjects, questions. I know I disagreed because I didn’t view them as high priority cases and they were not life threatening. I decided that we shouldn’t indulge in that until we sort out the serious cases. If that’s sorted out, then we should. Other group members, they also disagreed, but they disagreed in a, let me put this in words, give me a second. They mentioned a lot of reasons why they should disagree with it.

Donrich: Can you give us the main reasons?

Participant 12: Yes, about, you know, certain skin colour, people, like, ok, let’s, homosexual people – like some of them might get killed in certain areas. Now if somebody were to agree with that question, and then they decide to make somebody gay or something, then it will be a bad thing because they will get killed in certain areas. I can't remember exactly, a hundred percent, the points.

Donrich: But, Participant 12, thank you. I see Participant 19 has her hand raised, she would very much like to jump into the conversation and add to what you’re saying. Over to Participant 19.

Participant 19: Yes, so we reached a consensus. We all disagreed. We said that this should not be allowed. This should not be legal because it is a serious violation of the child’s ability to choose and they could, the child could feel extremely violated if they end up with hand-picked qualities that they dislike, so I feel that’s been mentioned by other groups as well. But then, with intelligence, we felt that I was taking away incentive. So, if somebody’s work ethic doesn’t have incentive and, life becomes boring, life becomes without purpose. So that was our main discussion – how working hard at improving your personality, how working hard to be less aggressive or more aggressive, you know, some of us are less assertive, some of us are more. So, working on those things gives you a sense of achievement and if you have been given those on a silver platter, there's no incentive. It takes away the incentive and therefore the purpose of life.

Donrich: Mm, mm.

Participant 12: Yes, the one point that I forgot to mention is that we will be in a boring world if everybody could customise themselves.

Donrich: We hear, we hear that. Participant 24?

Participant 24: Yes and also, there was also the question that if we increase intelligence and everybody is intelligent, aren’t we creating a society where nobody will learn from the other because everybody will feel that, ‘I'm intelligent enough, I can figure it out by myself’? so will that not create that kind of society? And, when it comes to personalities, are we not creating a society that is less accepting of others when we use gene editing to change some of the personality traits? So those are some of the points that we also came across.

Donrich: Thank you, Participant 24. Let’s move to my group. If I may ask Participant 9 to report back please.

Participant 9: So, I will just, I think I'm going to start from number 2 going downwards. I will get to number 1 lastly because we had the discussion. So, with personality traits, I think, I don’t think there was anyone who disagreed. I think pretty much agreed that it’s something that we shouldn’t edit at all. So, various reasons came up and I said that there is certain personality traits that you need for certain things in life, such as, in terms of having a child who is very cooperative, how then does that person be able to, basically, think for themselves? So, basically, it’s almost something of anything that they are told they must do, we know that some of the best things that have been discovered in life have come from going totally against everything that we have been taught. With sexual orientation, we disagreed with this one and we, the reasoning is that, already there is, there is quite a number of sexual orientations and we don’t think that it’s likely that humanity would suddenly want to converge back to only having basically the one or the two sexual orientations. So, we think that it’s something that as the child grows, they’ll be able to learn for themselves and to be able to pick what they like, what they don’t like, and what they would like to be in terms of sexual orientation. And then, lastly, sorry, the second last, so in terms of aesthetic characteristics, we disagreed with this one. We said it’s something that we think should not be touched. Participant 20 actually brought up an important point because I had raised the issue that, if, say you decide you want your child to have curly hair and you end up in an environment where everyone else has got, say, straight hair, they might, you know, feel like they are not fitting in. Participant 20 raised the point that, so now imagine if they then move to a different environment where everyone else there also has different hair texture from them, then does it mean that they should also then again change their hair texture? So I think it’s something that we should just leave up to our natural DNA and take it from there. And then, lastly, the most important one which was intelligence. Important points came up here. I think I still disagree but I’d like to raise the points that were brought up. So, mainly, something that came up was that generally, obviously a higher IQ does generally correspond to someone having better chances of success in life. I do agree with that but, and then also, just the fact that with higher IQ, or rather, more intelligence, we’ll probably be more innovative and come up with, you know, better discoveries or developments. But, I am inclined to disagree at some point because I think intelligence also affects decision-making. So, it could be for the better, but I also think it could be for the worse. We could end up in a situation where everyone almost has the same decision-making process, so then there's almost no, there's no room for dialogue, you know. It’s almost like everyone is in line with one thing.

Donrich: Participant 9, thank you very much. It was definitely a very brave effort from you to try to give both the perspective from our long conversation on intelligence. Thank you very much. Would anybody like to add to that at this stage? [silence] In that case I am moving on to Bongi’s group. If I may ask Participant 7 to report back for us.

Participant 7: Hi, thank you, yes. Well, we spoke about, most of us agreed on intelligence being, as far as I remember, being agreed, consensus on that. And then…

Donrich: So if I understand correctly, so in other words, that parents can have the choice, is that correct?

Participant 7: Yes. And then disagree on two, three and four. And then we made, we came, we were speaking about, I know Participant 5 made the point of South Africa should only have laws in place for the good of the child and not for cosmetic purposes. And she said also that if you wanted to go to another country and have these processes done, that you should have the right to do that. So, that was, yah, then we were also talking about, well, I was talking about it, we were speaking about, yes, the unknown effects of increasing intelligence. What could be the knock-on effect? I think other groups have spoken about it. We did speak about, for instance, cults or religious groups, you know, performing eugenics in certain ways, in certain, you know, it could be a very weird situation. You know, you’d get like cis-gendered because, and then the point was made that we are not a theocracy so state and religion are separated. Point was made by Participant 6 which I thought was part of our Constitution that we have to remember. We basically came to consensus, it was, you know, the other breakaway groups had been and there was no time left but we basically did come to that consensus. If anybody wants to add, thank you.

Donrich: Thank you very much, Participant 7. Any additions to that, from Bongi’s group? [silence] Great, well then that was our final report. The last opportunity for anybody who think that their positions have not been sufficiently, or their reasons has not been stated sufficiently in the plenary? Participant 3?

Participant 3: Yes, also I just wanted to add the part about drawing parallels between editing and biracial people as well as transracially adopted children. So if you look at today’s date, I mean today’s climate, most of people who are biracial or transracially adopted, they do not have a firm sense of belonging and they can find that they are rejected should they choose to identify strongly with one party that they were brought up by. So even though it was pointed out that, yes, a child can adopt or can choose to associate themselves strongly with whatever characteristics, whatever culture that they were brought up in, at the end of the day they still have to interact with the outside world so I believe that gene editing it will be the same issue again and we have not…

Donrich: With all four of the questions or specific ones of them? So, for instance will that affect the intelligence one, or will it only affect the traits? Just explain that to me.

Participant 3: So, with, I believe with intelligence and athleticism, I don’t feel so strongly about it, as well as, one and two I don’t feel so strongly about. Three, maybe that’s where I was like getting into because if I can just add on that. If, let’s say, I say I don’t want a child who is heterosexual, and then if I do get that child, how do you think I'm going to treat them? I'm going to reject them, punish them, and they’re going to have trauma, right? So, if we are saying we don’t want to have someone who has Down's syndrome, or autism, if we’re saying we’d like to fix them so that we can improve their quality of life, then how is this different? I am asking, I am not saying it’s the same. I am asking, how is this different? Because here it is like we are all saying oh no, it’s fine, but with serious illnesses on the previous days we were saying, yah, no quality of life will be improved. But now, when it comes to a possibility of being rejected, trauma, and so on, we’re just saying hmm.

Donrich: Ok, there are two hands, thank you very much, Participant 3. That’s definitely a novel explanation or perspective. Three hands are raised and I would like each one of you to be very succinct, very concise. Participant 15, Participant 22 and then Participant 12. Participant 15 first please.

Participant 15: I would like to take this opportunity in thanking y’all for allowing me this opportunity to partake in such an important discussion that could potentially reshape our world. I am extremely grateful to be given an opportunity to be able to have a say in our future as human beings. I have learned a lot during these deliberations. My understanding of concepts regarding gene editing has been broadened thanks to the debates we had over the past three days. My mind has been changed on many aspects that I would previously never have considered. Thank you again.

Donrich: Thank you and we are honoured by your participation, well, by the participation of all of our participants. Thank you, Participant 15. Participant 22, over to you.

Participant 22: I just wanted to say that I view the two situations quite differently, that most of the health issues that were raised, for me at least, I was agreeing because of how those will affect your health directly and changing someone’s sexual orientation so that someone else doesn’t treat them badly is, it’s not directly going to affect their health, it’s an indirect thing caused by someone else, not caused by their sexual orientation directly. So I feel like, it’s not, it would be much more effective to address the problem head on than to change the sexual orientation of the person. Rather spend the time and resources that you’d spend changing their genes to instead address the parents, give them counselling, make them understand that being heterosexual or homosexual or bisexual or whatever it may be is not a choice on the child’s part. Stop getting them to mistreat the child instead of changing the child’s sexual orientation.

Donrich: Participant 22, thank you very much for that response. Participant 12, over to you.

Participant 12: Hey. I would just like to say that these three days of deliberation have been really engaging. I have certainly changed my mind on a lot of aspects. But what I would like to point out is what we deliberating about, we’re just like reading about it and all, we've never seen it in actual, we’re not sure like, how it will actually look in the real world. We’re just believing everything we’re hearing. That’s it, thanks.

Donrich: Well, the technology is real, whether anything that we are discussing is already possible, that’s a different question. But gene editing is real and we appreciate everybody’s interaction regarding this. Any final points? Participant 22, is that an old hand or a new hand?

Participant 22: Yah, it was a new hand. May I just say that I do believe quite recently, I think two or three years ago a scientist in China was actually arrested as he edited two girls’ genomes to be more resistant against AIDS. So, I guess, it’s started already. There are people out there with genetically edited genomes.

Donrich: Indeed, indeed. That is indeed a fact, so it has already been done on humans to, in an attempt to make them immune against HIV/AIDS. Good. Participant 12, is that a new hand or an old hand?

Participant 12: A new hand. About that Chinese experiment. Yes, it’s still too early to say, I've heard that they are only two years old. We don’t know what future issues they might have in their lives, being genetically modified.

Donrich: Yah, indeed. I think what’s immensely important is that, generally, all over the world, what happened in China was judged as unethical because of, well, various reasons, but before we do anything, before we implement any new medical technology or medicine, there must always be proper clinical trials. Well, preclinical trials then clinical trials to establish that the new medicine or technology is, the new medical device, whatever, is safe and effective and that was not done in that case in China. So, that is why when we designed this study, we very specifically said at the beginning of all the questions that we, for purposes of this study, we assume that the gene editing that we are now contemplating in the future, will be safe and effective. In other words, it will only be after proper clinical trials to establish its safety and efficacy. Good. Participants, let’s do our final poll. In a second, you will have it in front of you. [silence] Michaela, if you can just advise me the number of participants currently online. I see that 24 people completed the poll at this stage, but I think there are 25, if you can just confirm.

Michaela: Yes, that’s correct, 25. So, we are waiting for one more.

Donrich: Ok for one person. There we go! We've got 25. Excellent, good. I will share the results with everybody. So there you see question 1, question 2, question 3 and question 4. Great well, facilitators, participants, we've come to the end of our deliberative public engagement event, at least the deliberations part. In a week’s time I would like to remind you that we will still ask you in a week’s time to do a post-deliberation survey because we want to see how you feel a week after the deliberations. So please don’t forget about that. Michaela will be in contact with everybody to ensure that everybody has… well Michaela might want to speak about that.

Michaela: Yes, I just wanted to say a couple people missed the poll tonight, I've seen a message in the chat, if you’re in here, and you didn’t manage to finish the poll, I will be sending it to you in a Google Form, so don’t stress about that.

Donrich: Thank you, Michaela. And Michaela will also be in contact with you. It’s very important that we give you at least fair compensation for participating in the research and Michaela will also be sorting that out. So, from my side, and from my fellow researchers who are part of this study, participants, thank you very much for investing your time and your effort and your emotion and your reason into our study. It’s very much appreciated and we will keep you abreast regarding publications so once we’ve got our articles and so on ready based on this study, we will let you know so you can also read it and then tell your friends, tell your family that you participated in this. So, on that good note, thank you very much and I wish you very well. Take care. Bye-bye.
